# Multiple introductions and recombination in *Cryphonectria hypovirus 1*: perspective for a sustainable biological control of chestnut blight

**DOI:** 10.1111/eva.12157

**Published:** 2014-04-15

**Authors:** Nicolas Feau, Cyril Dutech, Jérémie Brusini, Daniel Rigling, Cécile Robin

**Affiliations:** 1 INRA, UMR1202 BIOGECOF-33610, Cestas, France; 2 University Bordeaux, BIOGECO, UMR 1202F-33400, Talence, France; 3 TAIGA-Lab, Forest Sciences Centre, University of British Columbia#3618-2424 Main Mall, Vancouver, BC, V6T 1Z4, Canada; 4 Department of Ecology and Evolutionary Biology, Earth and Marine Sciences Building, University of CaliforniaSanta Cruz, CA, 95064, USA; 5 WSL Swiss Federal Research InstituteCH-8903, Birmensdorf, Switzerland

**Keywords:** biological control, disease biology, host parasite interactions, invasive species, microbial biology

## Abstract

*Cryphonectria hypovirus 1* (CHV1) is a mycovirus which decreases the virulence of its fungal host *Cryphonectria parasitica*, the causal agent of chestnut blight recently introduced in Europe. The understanding of the evolutionary processes which have shaped CHV1 populations in Europe is required to develop a sustainable biocontrol strategy targeting chestnut blight and effective in European chestnut forests. To retrace the evolutionary history of CHV1, we analyzed sequences from two genomic regions on a collection of 55 CHV1 strains from France and northern Spain, two countries where multiple introductions of *C. parasitica* occurred. Several recombination events and variable selection pressures contributed to CHV1 evolution, agreeing with a non-clock-like diversification rate. These two mechanisms may be at the origin of CHV1 population diversity observed in western Europe. Considering the actual prevalence of CHV1 and its association with host genotypes, multiple introductions of CHV1 may have occurred in Europe, some of them directly from Asia and some of them through North America. Although some viral strains remained with low frequency in their introduction area, multiple infections might have allowed homologous recombination within parental sequences. Some of these recombinant lineages are associated with the spread of CHV1 in European regions.

## Introduction

There is now growing evidence that global change could boost chronic impacts of plant pathogens, for example through climate warming that releases constraints on their development or through transport of exotic species (Pautasso et al. 2012). This prediction tends to be confirmed in forests where several new tree diseases emerged during the two last decades. In this context, biological control appears to be a suitable strategy to contribute to sustainable forest management. The use of natural enemies to regulate pathogen populations and to maintain them under an acceptable economic threshold of damage can be reached by different methods: the classical method is based on the release and establishment of non-native natural enemies of exotic pathogens, whereas methods of augmentation and conservation aim at increasing the effectiveness of natural enemies already present in the environment (Eilenberg et al. 2001). Mycoviruses, which decrease fungal virulence, are seen as promising agents to mitigate fungal tree diseases. Such viruses, infecting and replicating in pathogenic fungi, are causing a fungal debilitation which results in an overall decrease in reproduction rates and/or vegetative growth, and are, thus, beneficial to the host plant (Pearson et al. 2009). For example, mycoviruses could contribute to the attenuation of disease outbreaks after pathogens are introduced in a new area, as it has been suggested for chestnut blight and for Dutch elm disease (Milgroom 1999). Although mycoviruses are widespread within the fungal phylogeny (Ghabrial and Suzuki 2009; Pearson et al. 2009), studies on genetic and phenotypic diversity remain limited to very few mycoviruses (Voth et al. 2006; Bryner et al. 2012), and we are far to be able to predict how a mycovirus, introduced in a ‘new’ host population, will evolve. As a consequence, the use of mycoviruses as biological control agents requires an understanding of their ecology and evolution (Roderick et al. 2012).

Several points need to be examined before predicting the success of mycoviruses as biocontrol agents. On one hand, a biosafe candidate for biocontrol must exhibit a limited host range, avoiding its spread over to unexpected host targets. Mycoviruses are thought to have evolved in close association with their hosts (Ghabrial and Suzuki 2009) and have a limited host range, most often limited to one host genus (e.g., Liu et al. 2003). On the other hand, biocontrol agents must be able to establish and spread within the targeted host population. With the exception of free viral particles (Yu et al. 2013), mycoviruses are generally transmitted by an intracellular process, ensuring successful spread within the host population. Intracellular transmission may involve either vertical transmission through fungal spores (Pearson et al. 2009) or horizontal transfer by the way of anastomosis and heterokaryosis events (Leslie 1993). As the success of anastomoses in fungi is controlled by the vegetative incompatibility gene system (i.e., *vic* or *het* genes; Saupe 2000), efficiency of mycovirus spread can be directly dependent on the interactions of these genes within the host population (Cortesi et al. 2001).

Some mycoviruses have been detected in large geographic areas, as illustrated by *Cryphonectria hypovirus 1* (CHV1) the type species of hypoviruses (Hypoviridae family) which infects the fungal pathogen *Cryphonectria parasitica,* the causal agent of chestnut blight around the world. Native to eastern Asia, its introduction to North America at the end of the 19th century resulted in the near elimination of American chestnut (*Castanea dentata*). In Europe, where *C. parasitica* jumped to a new host (the European chestnut, *C. sativa*), several introduction events have occurred since the beginning of the 20th century, directly from Asia or via North America (Robin et al. 2009; Dutech et al. 2010, 2012). The low impact of *C. parasitica* in Europe could be partly explained by the introduction of CHV1 (Milgroom and Cortesi 2004). The typical ‘hypovirulence’ phenotype, induced by CHV1 in *C. parasitica*, encompasses several changes in the biology and physiology of its host and results in the survival of trees infected by such virus-infected fungal strains (Shapira et al. 1991; Nuss 2005). Hypovirulent *C. parasitica* strains were isolated for the first time in 1964 in Italy and then in France. CHV1 was later detected in *C. parasitica* in Japan and China where it had most likely originated (Peever et al. 1998). CHV1 is now detected in almost all European regions where *C. parasitica* was introduced, but so far has never been detected in American *C. parasitica* populations, except in a few sites where it has been intentionally introduced (Milgroom and Cortesi 2004). CHV1 is grouped within positive stranded RNA viruses and has a 12 712 nts genome which contains two open reading frames (ORFs) encoding multifunctional polyproteins (Nuss 2005; Ghabrial and Suzuki 2009). Based on variation found within both ORFs, five different subtypes have been characterized with sequence divergence levels ranging from 11% to 19% (Gobbin et al. 2003). Subtype I is widespread in southern and southeastern Europe from southeastern France to Turkey (Sotirovski et al. 2006; Robin et al. 2010; Krstin et al. 2011). Phylogeny and time estimates suggest that CHV1 was introduced into Italy together with *C. parasitica* and spread across south-central Europe and then eastern Europe (Bryner et al. 2012). By comparison, the distributions of the other subtypes are much more geographically restricted. Subtypes F1 and F2 were first found in France and recently in Spain (Zamora et al. 2012) and Turkey (Akilli et al. 2013); subtypes E and D were detected in Spain and Germany, respectively (Gobbin et al. 2003; Peters et al. 2012).

Very soon after the discovery of hypovirulent isolates, Grente and Berthelay-Sauret (1978) demonstrated that inoculation of cankers by compatible hypovirulent isolates resulted in canker healing. They suggested using a yearly release of hypovirulent strains as a treatment against chestnut blight in orchards. This biological control method has been successfully applied in southern France for 40 years (Grente and Berthelay-Sauret 1978). However, biocontrol with CHV1 still needs to be improved for reducing the density and impact of *C. parasitica* populations without continuous human assistance. Indeed, after an experimental release of hypovirulent strains in northern Switzerland where no hypovirulence was detected, CHV1 subtype I quickly established and spread (Hoegger et al. 2003), whereas in southern France, introduced subtype F1 was outcompeted by naturally occurring subtype I strains (Robin et al. 2010). Variability in fitness components within CHV1 strains and genotype × genotype × environment interactions might affect the outcome of biocontrol (Peever et al. 2000; Brusini 2009; Robin et al. 2010; Bryner and Rigling 2011). Moreover, CHV1 transmission processes via spores and distance of migration as well as transmission between vegetative groups are far to be understood in natural populations. Asexual spores, in which CHV1 is vertically transmitted, have short distance dispersal. Ascospores, which are wind dispersed, have never been found infected by CHV1 (Carbone et al. 2004; Prospero et al. 2006). Phylogenetic studies (Carbone et al. 2004) and experimental *in planta* assays (Brusini and Robin 2013) indicated that the ability of CHV1 to disseminate among fungal populations composed of several vegetative compatibility (vc) types might have been underestimated by *in vitro* studies, suggesting that heteroallelism at *vic* loci strongly reduced virus transmission (Cortesi et al. 2001). There is also a need to expand biological control to forest plantations and coppices and to European areas where chestnut blight recently emerged but where CHV1 did not yet established, for example northern France (De Villebonne 1998), Portugal (Bragança et al. 2007), northern Switzerland (Hoegger et al. 2000) and part of southwestern Germany (Peters et al. 2012). In these regions, chestnut blight impact is high and the development of a sustainable biological method has been requested by stakeholders.

The primary objective of our study was to assess the genetic diversity and structure of populations of CHV1 sampled in a geographic area where multiple introduction events were detected in *C. parasitica*. Our hypothesis is that genetic structure of the mycovirus is highly spatially correlated with the introduced host populations. The second objective was to study evolutionary processes (recombination, mutation, migration and selection) which could generate or maintain variation in CHV1 occurring in Europe. New viral genotypes can be created through recombination events after multiple introductions and be beneficial for biological control of chestnut blight. So far, evolutionary changes have only been inferred from areas where only one CHV1 subtype (subtype I) occurs (Bryner et al. 2012). To understand the evolutionary history of CHV1 in Europe, investigations were carried out in areas with higher CHV1 subtype diversity and different host populations.

## Materials and methods

### Fungal isolates

**Figure 1:**
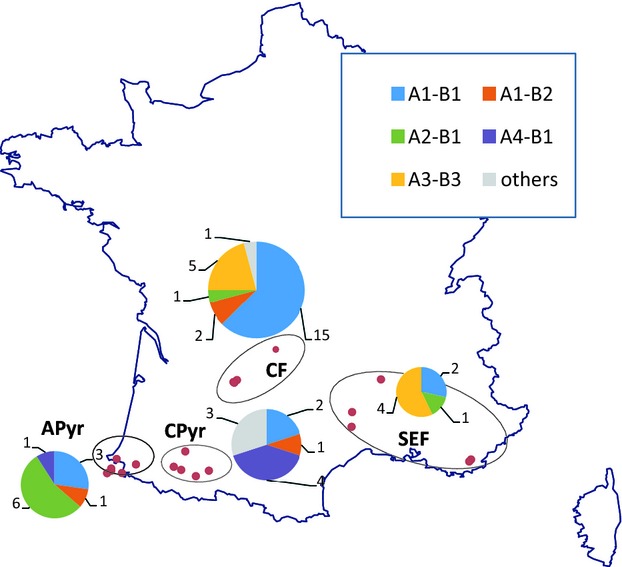
Sampling localities of *Cryphonectria hypovirus 1* strains in four regions: southern-eastern France (SEF), Central France (CF), Central Pyrénées (CPyr), and Atlantic Pyrénées (APyr). Pie charts represent the frequency of the main viral lineages (A1B1, A1B2, A2B1, A3B3, A4B1; one color for each, all other lineages are represented in grey) in the four regions.

Isolates used in this study were obtained from untreated cankers during previous surveys for *C. parasitica* (Robin et al. 2000; Breuillin et al. 2006; Dutech et al. 2008; Robin et al. 2009; Dutech et al. 2010). From this collection, we analyzed 49 fungal isolates exhibiting the white phenotype (reduced pigmentation and sporulation) usually associated with a CHV1 infection: five isolates were from southeastern France (SEF), 24 isolates from central France (CF), 11 isolates from central Pyrénées in France (CPyr), and nine isolates from the Atlantic Pyrénées in Spain and France (APyr) (Fig. 1, Table 1). In addition, five CHV1 strains (named as [fungal isolate]), previously assigned to subtype F1 [(2091) and (48.2D)], subtype F2 [2103], and subtypes E and D (strains [M1147] and [M1372]) were included in our sample (Gobbin et al. 2003). Finally, this heterochronous sampling of CHV1 strains was completed with data from two isolates available in Genbank: the complete genomes of the viral strains [EP713] (Shapira et al. 1991) and [Euro7] (Chen and Nuss 1999). EP713 is an American *C. parasitica* isolate (EP155) infected by the hypovirus that was originally obtained in 1966 from southeastern France (Robin et al. 2010; S. Anagnostakis, personal communication). Euro7 is a naturally virus-infected isolate originating from Italy. Forty-five fungal isolates were previously genotyped with 10 microsatellites markers. They could be assigned to one of the three genetic clusters defined among 105 French genotypes known so far (Dutech et al. 2010). Nineteen isolates (from southeastern and central France) were grouped into the genetic cluster G1, 20 and 4 (from the Pyrénees area and central France) belonged to cluster G2 and G3, respectively.

**Table 1:** Origin and genetic clusters of *Cryphonectria parasitica* (*Cp*) isolates infected by *Cryphonectria Hypovirus 1* (CHV1), viral lineages and recombination event detected with RDP3 within two genomic regions of CHV1

			GPS coordinates (Lambert II)					
							
*Cp* isolate	Region	Locality	X	Y	Year of sampling	*Cp* genetic cluster*	CHV1 genetic cluster	Recombination event†
48.2D	SEF	Gabriac	711 009	1 909 572	1997	G1	A1B1	E1
Gan20	SEF	Ganges	709 931	1 882 176	1998	G1	A3B3	E2
Gan32	SEF					G1	A3B3	E2
07.4A	SEF	Genestelle	763 047	1 970 867	1995	.	A3B3	E2
Gon37	SEF	Gonfaron	92 1018	1 821 194	1999	G1	A2B1	E5
2145	SEF	Les Mayons	926 478	1 820 660	1977	G1	A3B3	E2
Ep713	SEF				1966	.	A1B1	E1
Doi1	CF	Doissat	501 563	1 969 760	2003	G1	A4B3	E4, E2
Doi6	CF					G2	A1B1	E1
Doi12	CF					G1	A3B3	E2
Doi15	CF					G1	A3B3	E2
Doi17	CF					G1	A3B3	E2
Doi21	CF					G1	A1B1	E1
Doi38	CF					G1	A1B1	E1
Doi40	CF					G1	A1B1	E1
Doi42	CF					.	A1B1	E1
Doi60	CF					G2	A1B1	E1
Doi74	CF					G1	A1B1	E1
Doi88	CF					G1	A1B1	E1
Doi94	CF					.	A1B1	E1
Maz9	CF	Mazeyrolles	495 533	1 966 028	1998	G3	A1B1	E1
Maz31	CF					G1	A3B3	E2
Maz34	CF					G1	A1B1	E1
Maz47	CF					G1	A1B1	E1
Maz49	CF					.	A2B1	E5
SC3.3	CF	St Cernin	496 966	1 964 707	2007	G2	A1B1	E1
SC36.4	CF					G1	A3B3	E2
SC61.3	CF					G2	A1B1	E1
SC77.1	CF				2005	G2	A1B2	NR
SC77.4	CF					G2	A1B2	NR
2091	CF	St Pardoux	599 393	2 068 258	1974	.	A1B1	E1
Arn11	CPyr	Arné	447 527	1 796 527	2002	G2	A1B1	E1
Arn17	CPyr					G2	A1B1	E1
Arn6	CPyr					G2	A4B1	NR
Bor14	CPyr	Bordes	387 260	1 809 382	1998	G2	A2B1	E5
Bor26	CPyr					G2	A2B1	E5
Bor31	CPyr					G2	A2B1	E5
Bor41	CPyr					G2	A2B1	E5
Bor42	CPyr					G2	A1B2	NR
Bor47	CPyr					G2	A2B1	E5
2103	CPyr	Castillon				.	A2B1	E5
Mon5	CPyr	Monpezat	408 103	1 836 029	2002	G2	A1B1	E1
Pon16	CPyr	Pontacq	400 422	1 801 220	2002	G2	A2B2	E3, E5
Lan4	APyr	Lantabat	318 534	1 811 481	2002	.	A4B1	NR
Lan6	APyr					.	A1B1	E1
Sar11	APyr	Sare	283 448	1 821 731	2002	G2	A1B1	E1
Sar15	APyr					G2	A4B3	E4, E2
Don2	APyr	Doneztebe	273 685	1 799 794	2002	G2	A4B1	NR
Don5	APyr				2002	G3	A4B1	NR
Lab7	APyr	Labaien	266 006	1 795 181	2002	G3	A5B2	E3
Sun9	APyr	Sunbilla	273 411	1 803 694	2002	G3	A5B2	E3
Val5	APyr	Valcarlos	30 2863	179 3868	2002	G2	A1B2	NR
M1147	APyr		Alleman et al. 1999		1998	G2	A4B1	NR
M1372	Germany	Oberkirch	Alleman et al. 1999		1992	G1	A4B2	E4
Euro7	Italy	Florence	Chen and Nuss 1999	1999	1978	.	A3B3	E2

SEF, Southeastern France; CF, central France; CPyr, central Pyrénées; APyr, Atlantic Pyrénées.

* Genetic clusters were defined in Dutech et al. (2010).

† NR: non-recombinant viral strain.

### DsRNA isolation, cDNA synthesis and sequencing

*Cryphonectria parasitica* isolates were grown on cellophane-covered PDA plates for 7 days at 25°C in the dark. The mycelium was scraped from the cellophane, freeze-dried and ground to a fine powder in a Hybaid Ribolyser (Hybaid, Teddington, Middlesex, UK). Viral dsRNA was extracted from these powders (20–50 mg) using a CF-11 cellulose chromatography method (Alleman et al. 1999). One microliter (approximately 50 ng) dsRNA solution was lyophilized together with 0.5 μg random primers (Promega, Madison, WI, USA) and cDNA synthesized as described by Allemann et al. (1999).

Two regions of the CHV1 genome were sequenced. The first one from ORF A (positions 1495–1792 in the nucleotide sequence of CHV1/EP713) amplified with primers hvep1 and hvep2 (Gobbin et al. 2003). For this region, sequencing of both strands was performed at the Swiss Federal Research Institute (WSL) using an ABI Prism 3130 Genetic Analyzer (Life Technologies, Carlsbad, CA, USA). The second one, from ORF B (positions 6252–6991 in the nucleotide sequence of CHV1/EP713) amplified with primers developed for this study: ORF B-12F (5′-ATCGGGTCTCCCTTCAAGTT-3′) and ORF B-12R (5′-CACGACGAGTTCGTTGAGRA–3′). In this case, sequencing of one strand was performed by GATC-biotech (http://www.gatc-biotech.com). The annealing temperature for amplification with the ORF A and ORF B primer sets were 55°C and 58°C, respectively.

Sequence nucleotide bases were checked for a minimum Phred quality score of ≥20, aligned and chromatograms were checked for any ambiguous nucleotides and then aligned using ClustalW (Thompson et al. 1994). The ORF A alignment was comprised of 298 bp. Although ORF B-12F and ORF B-12R amplified a cDNA fragment of about 780 bp, the flanking sequences were removed and only 591-bp sequences were used for analyses of this genomic region.

### Phylogenetic analyses

For each ORF, base content, chi-squared test of homogeneity of base frequencies across sequences and distances were computed, and phylogenetic relationships among haplotypes were inferred using maximum-parsimony (MP) method with PAUP ver. 4.b10 for PC (Swofford 2003). MP trees were generated through heuristic searches with 1000 random stepwise additions, with tree bisection-reconnection branch swapping and saving multiple parsimonious trees (MULTREES turned on). Nonparametric bootstrap support values for each dataset were estimated by performing 1000 heuristic searches with 100 random stepwise additions and all other settings as above. The homoplasy matrices generated in PAUP were visually inspected to locate parts of trees where there was phylogenetic conflict and to identify the haplotypes responsible for this conflict.

### Recombination analysis

A phylogenetic network was constructed from the concatenated alignment of ORF A and ORF B. The network was obtained by the neighbor-net method implemented in SPLITSTREE v4.11.3 (Huson 1998). To confirm the occurrence of recombination within CHV1, localization of recombination breakpoints and identification of likely parental sequences were carried out with RDP3 (Martin et al. 1998). Several recombination detection methods are implemented by this program: RDP, Geneconv, Bootscan, Maxchi, Chimaera, Siscan and 3seq. Default settings were used throughout. Firm recombination patterns were those detected by seven different methods, after Bonferroni correction for multiple comparisons. The likely parental sequences are the non-recombinant sequences which had regions the most similar to recombinant sequences.

### Estimation of divergence dates

Analyses were conducted on subsets of ORF A and ORF B alignments (ORF A_sh_ and ORF B_sh_) including only non-recombinant sequence blocks to avoid recombination bias. The molecular clock hypothesis was first tested by using the likelihood ratio test (LRT) to compare the null hypothesis of a molecular clock to the alternative hypothesis that lineages evolve at different rates (Hasegawa et al. 1985). The HKY model was the best model for both data subsets using the Akaike information criteria (AIC) in jModelTest ver. 0.1.1 (Posada 2008). Second, ‘clock-likeness’ of the ORF A_sh_ and ORF B_sh_ phylogenetic signals was assessed with the Path-O-Gen software (http://tree.bio.ed.ac.uk/software/pathogen/) by calculating the coefficient of variation (CV) of distance (*i.e.,* the standard deviation to the mean ratio of the distances) from the tree's ancestral root to each of the terminal branch tips. The CV was taken as an indication of how clock-like the evolution was: the lower the coefficient, the more clock-like the evolution. Third, the marginal likelihoods of the strict and of the relaxed clock models (uncorrelated lognormal and exponential models) were compared within a Bayesian coalescent framework by a Markov Chain Monte Carlo method using the Bayesian Evolutionary Analysis by Sampling Trees program BEAST (http://beast.bio.ed.ac.uk; Drummond and Rambaut 2007) utilizing the HKY model of nucleotide substitution and a prior substitution rate of 2.56e^−05^ substitutions/site/year (95% CI, 0.0–1.70 x 10^−4^) as determined previously for CHV1 (Gobbin et al. 2003). The best fitting model was selected by calculating the Bayes Factors (log_10_(BF) using the method developed by Drummond and Rambaut (2007) and described in Data S1.

### Identification of most probable location state for lineages

To determine the probable geographical origin of the different CHV1 lineages detected in the six sampling regions, we used a model of diffusion to discrete states to infer maximum clade credibility trees (Lemey et al. 2009). We first assigned sequences from the same approximate region (based on sampling geographical coordinates) to one of the six discrete sampling regions by using the R-libraries ‘geosphere’ and ‘cluster’ (Table S1 for details). Then, based on the results obtained in the previous clock-model analyses, continuous-time models were calibrated for the ORF A_sh_ and ORF B_sh_ datasets under a constant population size tree prior and an exponential relaxed molecular clock. Individual BEAST runs were performed with 200-million steps in the Markov chain and sampling every 10 000 steps to produce a posterior tree distribution containing 20 000 genealogies. The maximum clade credibility tree (a point estimate of the tree with the highest cumulative posterior probabilities in the posterior distribution of trees) was annotated with geographical locations using the software Tree-Annotator (available in BEAST package). For each node with a bayesian posterior probability higher than 0.5, posterior state probabilities (PSP) were assigned to the six sampling populations.

To avoid bias arising from unequal sampling size between locations, we homogenized the sample sizes for all locations from which more than eight sequences had been sampled, by randomly sub-sampling eight sequences from each of these. Sequences from Italia and Germany with only one sequence in each were discarded for these analyses. For each of the 25 smaller datasets constructed, we performed the same BEAST-phylogeographic analyses as those described above. Each time a tree node was found being supported with a bayesian posterior probability ≥0.5, PSP for each of the four sampling region (central France, Center Pyrénées, Atlantic Pyrénées and southeastern France) were collected. This resulted for each node in four distributions of probability values that were compared by using an one-way anova, and a Tukey's HSD test (after) was performed when the sampling region effect was significant. Finally, to obtain a greater confidence, the ORF A_sh_ dataset was enriched with additional sequences from Italy (*n* = 3), south of Switzerland (*n* = 15) and France (*n* = 2) previously published (Gobbin et al. 2003). Clock-model and phylogeographic analyses were carried out with this larger dataset as described above.

### Positive selection

To test for positive selection in ORF A_sh_ and ORF B_sh_ subsets, we estimated the rate of non-synonymous nucleotide substitutions per non-synonymous site (d_N_), the rate of synonymous nucleotide substitutions per synonymous site (d_S_) and the ratio *ω* = dN/dS, with the method of (Nielsen and Yang 1998) implemented in the PAML package of the computer program CODEML (Yang 1997). PhyML ver. 3.0 (Guindon et al. 2010) was used to estimate the branch lengths of the phylogenetic trees of the subset phylogeny, which were used as starting values for CODEML. Two models of codon substitution (M2a and M8) that allow for heterogeneous selection pressures among sites were compared to null models (homogenous rate among sites, models M1a and M7) as described in Data S2.

## Results

### Sequence analysis

We obtained unambiguous sequences for ORF A and ORF B for all but one viral strain ([Doi9]). The ORF A sequence of this strain could not be analyzed because of mixed sequence data. To test the hypothesis of an infection by more than one viral strain, mono-conidial isolates of this fungal culture were grown, and CHV1 strains infecting this progeny were analyzed. Two different viral strains were detected: one related to [EP713] and one related to [Euro7], confirming the double infection hypothesis of the Doi9 isolate. This isolate was then excluded from the analyses. We constructed an alignment of the 55 partial sequences of the ORF A and ORF B genomic regions of CHV1, including sequences retrieved from the NCBI database.

In the ORF A region (298 nt), 112 sites were polymorphic giving a rate of 0.384 substitutions per site. Among the 55 sequences, 40 haplotypes were identified. The most frequent haplotype was detected in seven fungal isolates: five from one location in central France (Doi6, −40, −460, −474 and −494) and two from the Pyrénées range (Lan6 and Arn11). Three other haplotypes were also found in fungal hosts from different geographic regions. In contrast, four other haplotypes were found in isolates originating from the same geographic location. In the ORF B region (591 nt), 166 sites were polymorphic and the substitution rate per site was 0.286 for the whole sample. Forty-nine haplotypes were defined from ORF B, with four haplotypes representing two or three viral strains. Two strains ([2103] and [Gon37]) shared the same haplotype for both ORFs.

### Phylogenetic relationships between CHV1 strains with maximum-parsimony analysis

The analysis of nucleotide frequencies over all codon positions suggested a compositional bias toward G+C bases. However, chi-squared tests of homogeneity of base frequencies across taxa were non-significant within 1st, 2nd and 3rd codon positions (Fig. S1). Of the three codon positions, third position nucleotides were systematically more variable than those of first and second positions as measured by percentage of sites that were variable and the percentages of sites that were parsimony informative (data not shown). To test the hypothesis of saturation of the phylogenetic signal at third codon positions, we calculated and plotted uncorrected distances and corrected distances between each pair of sequences. For each dataset, high slopes (above 0.65, except for first codon positions in ORF B) coupled with significant linear relations (all *r*^2^ ≥ 0.97) indicated little saturation at these codon positions (Fig. S1). Thus, nucleotide composition and saturation analyses showed that uninformative data should not adversely affect the phylogenetic reconstruction analysis (Fig. S1).

Maximum-parsimony analysis with heuristic searching in PAUP resulted in 150 and 105 trees for ORF A and ORF B respectively, from which consensus trees were built (Fig. S2). Using only the first and second codon position (*i.e.,* discarding positions that could had been subjected to multiple substitutions; see above), the topologies and resolutions reflected those obtained for all codon positions (data not shown).

For ORF A, five main clusters were resolved and supported with bootstrap values ≥0.85 (Fig. S2, Table 1). Viral strains defined as subtypes F1 ([EP713], [2091] and [48.2D]), F2 ([2103]) or I ([Euro7]) were enclosed within cluster A1 (26 strains), A2 (nine strains) and A3 (10 strains), respectively. However, strains [M1147] and [M1372] (subtypes E and D, respectively) grouped in one cluster (A4), with six other viral strains. The fifth cluster (A5) was represented by a unique viral haplotype, infecting two fungal isolates from the Navarre province in Spain (Sun9 and Lab7).

In ORF B, only three clusters, supported by strong bootstrap values (100%), were resolved (Fig. S2, Table 1). All strains that clustered together in A1 and A3 also grouped together in clusters B1 and B3, respectively. However, there were inconsistencies between ORF A and ORF B phylogenies. The A2 strains clustered with B1 strains, and the A4 strains ([Sar15] and [Doi1]) clustered in B3. The A5 strains ([Sun9] and [Lab7]) were regrouped with four A1, one A2 and one A4 strains in the B2 cluster.

Visual inspection of the homoplasy matrices generated in PAUP for the ORF A and ORF B MP trees helped to identify putative recombinants (Fig. S2). In each dataset, the distributions of homoplasy values were trimodal with values around zero, indicating little phylogenetic conflict, and higher values in the range of 10–20 and >45, indicative of more conflict. Similar patterns of phylogenetic conflict were found when the codon positions potentially saturated (third position) were discarded from the analyses (data not shown). In ORF A, conflicts were found between taxa of the A4 and A1 groups (homoplasy values ranged between 10 and 18), A1 and A2 groups and A1 and A4 groups (values between 46 and 56). Similarly for ORF B, values ranged between 70 and 90 indicating conflicts between taxa of the B1 and B2 groups.

### Recombination and definition of CHV1 main genetic clusters

**Figure 2:**
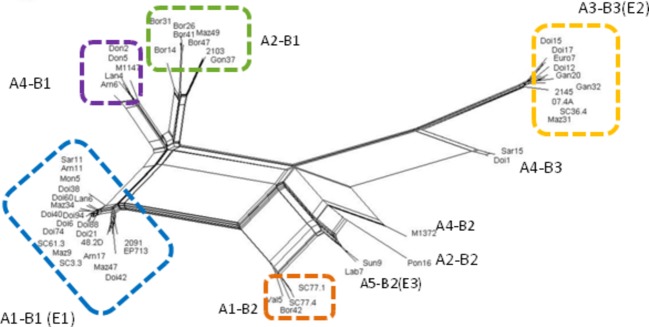
The phylogenetic network resulting from the split decomposition of concatenated sequences from ORF A and ORF B of *Cryphonectria hypovirus 1* using the neighbor-net method implemented in SPLITSTREE v4.11.3.

The concatenation of the two ORF regions and the split decomposition analysis of the concatenated sequence resulted in a phylogenetic network, in which the sequences were linked to each other by several branches suggesting a complex recombination history (Fig. 2). Viral strains with B1 sequences were clearly separated from strains having B2 and B3 sequences (Fig. 2). Parallel edges were also depicted betweens strains A1 and A4 and A5. Five main clusters gathered 89% of the concatenated sequences (clusters A1-B1 represented by 22 strains, A3-B3 10 strains, A2-B1 8 strains, A4-B1 5 strains and A1-B2 4 strains, Table 1).

**Figure 3:**
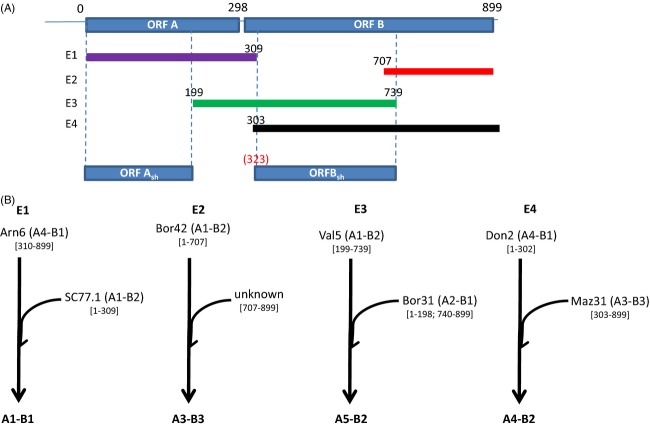
(A) Firm recombination events detected (E1, E2, E3 and E4) within 55 viral sequences of *Cryphonectria hypovirus 1* by using seven methods of the RDP3 software. (B) Putative scenarios of the recombination events with putative parental and resulting recombinant CHV1 lineages identified by split decomposition (cf Fig. 2).

Four firm recombination events were detected with the RDP3 program (Table S1, Fig. 3). They resulted in 38 recombinant strains (out of 55, i.e., 69%). The two main clusters identified with the phylogenetic network (A1-B1 and A3-B3) gathered recombinant strains resulting from the events E1 and E2 (Fig. 2). A1-B1 strains were mainly located in central France and in the Pyrénées region (20 strains among 22). A striking result is the absence of A3-B3 strains from the western part of the study area. In sequences from clusters A2-B1, A1-B2 and A4-B1, no firm recombination pattern was detected. Therefore, all these strains, coming mainly from the Pyrénées, were considered non-recombinant strains.

Among the 45 fungal host isolates assigned to one of the genetic pools defined for *C. parasitica*, 39 were infected by viruses belonging to one of the five main lineages. The relationship between viral and host genotypes was statistically significant (Fisher's exact test: *P* = 0.044e^−6^, two sided Probability = 0.0005). All viral strains belonging to the lineage A3-B3 were found in the fungal G1 gene pool and all A1-B2 strains in the G2 gene pool (Table 2). By contrast, A1-B1 viral strains were found in all three fungal pools.

**Table 2:** Distribution of CHV1 lineages in its host (*Cryphonectria parasitica*) genetic clusters

	Viral lineages†					
	
Host genetic clusters*	A1-B1	A3-B3	A2-B1	A4-B1	A1-B2	Others
G1	8	8	1			2
G2	8		5	3	4	2
G3	1			1		2
ND	5	2	2	1		0

* Defined in Dutech et al. (2010), ND: not determined.

† Lineages defined after concatenation of ORF A and ORF B sequences.

### Estimation of divergence dates

**Figure 4:**
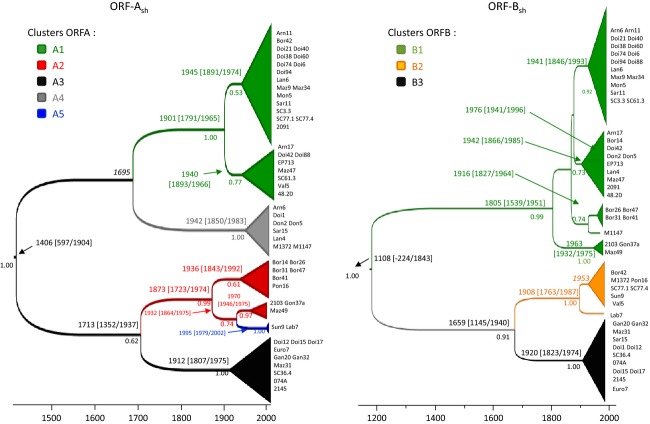
Lineages divergence dates inferred from ORF A_sh_ and ORF B_sh_ sequences of *Cryphonectria hypovirus 1*. The time-scale of evolutionary changes is indicated by the scale bar below the tree. Divergence dates with 95% CI are indicated above nodes; Bayesian posterior probabilities up to 0.5 are indicated below nodes.

Based on the recombination events previously identified, two non-recombinant data subsets (ORF A_sh_ and ORF B_sh_) were reconstructed (Fig. 4). LRT-tests rejected the null hypothesis of a homogeneous rate of evolution among lineages for the two subsets (Table 3). For ORF A_sh_, rejection of the null hypothesis was supported by a high degree of deviation from the strict molecular clock model in the root-to-tip test (CV = 0.16) and a significantly higher marginal likelihood value for the relaxed uncorrelated exponential molecular clock (log_10_BF >1.3 when compared to strict clock model; Table 4, Table S2). For ORF B_sh_, deviation from the strict clock model was less clear. On one hand, the low CV value of 0.10 and a highly significant LRT test did not indicate a clock-like evolution of this subset. On the other hand, differences in the marginal likelihood value between the strict and relaxed molecular clock models were below the significance threshold of 1.3 generally accepted (log_10_BF = 0.49, Table 3).

**Table 3:** Molecular clock model fitting for the ORF A_sh_ and ORF B_sh_ regions of *Cryphonectria Hypovirus 1*

			LRT test†			Molecular clock model‡			
					
Region	Length	Root-to-tip CV*	−Ln H_0_	−Ln H_1_	*P*-value	Strict	Relaxed exponential	Log_10_ BF	Model selected§
ORF A_sh_	199	0.16	910.45	766.07	<0.0001	−760.1	−756.4	1.62	Relaxed exponential
ORF B_sh_	383	0.043	1525.64	1351.98	<0.0001	−1392.8	−1389.1	1.58	Relaxed exponential

* Coefficient of variation that is ratio of the standard deviation to the mean for the root-to-tip distances.

† Likelihood ratio test between likelihood values obtained under the null hypothesis of a molecular clock and the alternative hypothesis each tree branch is allowed to vary independently.

‡ Likelihood values of the BEAST molecular clock models (strict molecular clock and relaxed exponential molecular clock) applied to the datasets. BF, Bayes Factor(log_10_) values between the likelihood values obtained for the strict and the relaxed molecular clock models.

§ Clock model selected for divergence time estimations.

**Table 4:** Maximum likelihood tests of positive selection into two genomic regions (ORF A_sh_ and ORF B_sh_) of *Cryphonectria Hypovirus 1* (CHV1)

CHV1 ORF	Model	Model parameters	-lnL	Models comparison	2ΔL	*Pr*.	No. sites under positive selection (Pr. *ω* > 1)
A_sh_	M1a (neutral)	*P*_*0*_ = 0.80*, P*_*1*_ = 0.20	714.73	M1a vs M2a	7.71	<0.01	1 (0.99)
	M2a (selection)	*P*_*0*_ = 0.86, *P*_*1*_ = 0.13, *P*_*2*_ = 0.096, *ω*_2_ = 6. 08	710.88				
	M7 (beta)	*P* = 0.008, *q* = 0.024	714.95	M7 vs M8	8.25	<0.01	1 (0.99)
	M8 (beta + *ω*)	*P*_*0*_ = 0.99, *P* = 0.26, q = 0.96, *P*_*1*_ = 0.010, *ω* = 5.71	710.82				
B_sh_	M1a (neutral)	*P*_*0*_ = 0.83*, P*_*1*_ = 0.17	1332.69	M1a vs M2a	7.16	<0.01	2 (0.99, 0.98)
	M2a (selection)	*P*_*0*_ = 0.83, *P*_*1*_ = 0.16, *P*_*2*_ = 0.005, *ω*_2_ = 5.81	1329.11				
	M7 (beta)	*P* = 0.005, *q* = 0.02	1333.07	M7 vs M8	8.25	<0.01	3 (0.96, 0.99, 0.99)
	M8 (beta + *ω*)	*P*_*0*_ = 0.99, *P* = 0.01, q = 0.08, *P*_*1*_ = 0.005, *ω* = 5.57	1328.95				

Three demographic model parameters (i.e., constant size, exponential size and expansion size) were also tested for each clock model fitted to the data subsets (Table S2). Marginal likelihood analyses showed that no other demographic model significantly fit ORF A_sh_ and ORF B_sh_ data better than the constant size population model [log_10_(BF) always <0.94], suggesting that there could be insufficient information in the data to fit a more complex demographic model.

Finally, using an exponential with constant population size model, average evolution rates were 2.483 x 10^−4^ [95% credibility intervals (CI) of 0.289 x 10^−4^ to 4.929 x 10^−4^] and 1.716 x 10^−4^ (95% CI, 0.303 x 10^−4^ to 3.410 x 10^−4^) substitutions per site per year for ORF A_sh_ and ORF B_sh_, respectively (Table S3). Under these rates, the tMRCA obtained for the different virus lineages with the two data subsets were mostly consistent, except for the age of the most recent common CHV1 ancestor [605 years for ORF A_sh_ (1406, 95% CI, −809/+498 years) and 903 years for ORF B_sh_ (11 088, 95% CI, −1332/+735)] (Fig. 4). The analysis of the full dataset (i.e., ORF A + ORF B), including interlineage recombinants with highly divergent CHV1 genes, resulted in an estimation of the time to the most recent common CHV1 ancestor of 110 years before (i.e., at 998 years; 95% CI, −1353/+816) the date estimated with the ORF B_sh_ dataset (Fig. S3).

Internal nodes differentiating sister CHV1 lineages (though generally weakly resolved; but see below) are dated between 1659 for the B2-B3 node (ORF B_sh_) to 1713 for A2-A3 (ORF A_sh_), with the 95% upper CI generally overlapping the first half of the twentieth century (1937 and 1940 for B2-B3 and A2-A3, respectively, Fig. 4). The tMRCA for clusters A3 and B3 (all strains A3 are B3) were established at 1912 and 1920, (with 95% CI, −105/+63 and −97/+51 years), respectively. Radiation times for the two lineages A1 (1945, 95% CI, −54/+29 years and 1940, −47, +26) and A4 (1942, 95% CI, −92/+41 years) fit with the tMRCA range for the corresponding strains within the B1 lineage (1942, −76, +43). Unfortunately, the phylogenetic resolution obtained for the MP consensus tree with ORF A for the A1/A4 and A2 clusters (bootstrap support of 100%) was not obtained with the BEAST analysis on the ORF A_sh_ subset, preventing dating the tMRCA for this cluster. Despite this, radiation of the A2 viral lineage was estimated at 1873 (95% CI, −150/+101 years) with this subset, 68 years (95% CI, −150/+101 years) after the tMRCA estimated for lineage B1 with ORF B_sh_ (Fig. 4).

### CHV1 lineages' most probable location of origin

**Figure 5:**
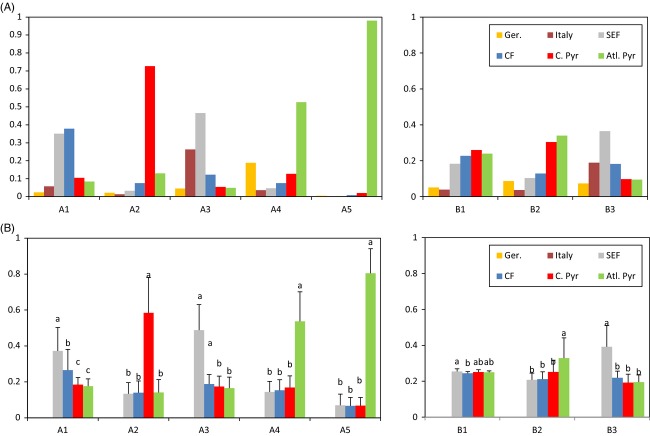
Posterior state probabilities of population origin (PSP) inferred from *Cryphonectria hypovirus 1* ORF Ash (left panels) and ORF Bsh (right panels) datasets. (A) PSP-values obtained for the original datasets. (B) PSP-values obtained after resampling ORF Ash or ORF Bsh datasets 25 times (see Methods). Letters above columns indicate significant differences among posterior location probabilities means as determined by a Tukey's HSD test after significant one-way anova.

Both ORF A_sh_ and ORF B_sh_ provided congruent information about putative origins of the different lineages, with generally a greater resolution for ORF A_sh_ (Fig. 5, Fig. S3). For the deepest nodes (i.e., those dated before 1850), similar PSP were generally assigned to the six sampling populations. Lack of phylogenetic structure at this level, consistent with the weak phylogenetic support observed, may explain the failure to clearly determine a probable origin for these nodes. Results from both ORFs converged in a way that the most recent common ancestor for lineages A3 and B3 likely emerged from southeastern France (PSP = 0.47 for lineage A3 and 0.37 for B3) or Italy (PSP = 0.26 for A3 and 0.19 for B3, Fig. 5). Re-analyzing ORF A_sh_ with 18 additional sequences from Switzerland and Italy also indicated an eastern origin for the A3 lineage (PSP = 0.63 for Switzerland/Italy and 0.26 for South–East; data not shown). A2 lineages likely originated from the Center Pyrénées (PSP = 0.73, Fig. 5) and A4 and B2 lineages from the Atlantic Pyrénées (PSP = 0.53 and 0.34, Fig. 5). Origin for the A1 strains was less clear with 35% of the trees assessed during the analysis consistent with an ancestral sequence originating from southeastern France and 38% from central France. Similarly, the probable origin of the B1 strains could not be fully resolved. ORF B_sh_ indicated a probable origin from southeastern France and central France for half of these strains (PSP = 0.26 and 0.33, respectively) and central and Atlantic Pyrénées (0.35 and 0.30, respectively) for the other half (Fig. S4).

### Positive selection

For both data subsets, LRT-values for comparing models M1a with M2a and M7 with M8 were statistically significant, suggesting that discrete models M2a and M8 systematically fit these two data subsets better than the neutral models M1a and M7 (Table 4). Model M2a indicated that 0.8% and 0.4% of the sites were under strong positive selection with *ω* = 6.08 and *ω* = 5.75 in ORF A_sh_ and ORF B_sh_, respectively. Model M8 showed for the two subsets that about 99% of the sites had *ω* from a U-shaped beta distribution, and about 0.5–1.0% of sites were under strong diversifying selection with *ω* = 5.78 for ORF A_sh_ and 5.58 for ORF B_sh_. In OFR A_sh_ under models M2a and M8, empirical Bayes' theorem identified one amino acid site under positive selection with >99% confidence. In ORF B_sh_, the two same sites under positive selection identified with model M2 (98 and 99% of confidence) were also identified with model M8 (>99% confidence).

## Discussion

Our sequence analysis of CHV1 strains from France and Spain revealed a high virus diversity and multiple introductions of viral lineages. This result markedly contrasts with the CHV1 diversity in southern and southeastern Europe, where only one CHV1 subtype (subtype I) was found (Bryner et al. 2012). Sequencing more than one genomic region was necessary to discriminate lineages as illustrated by subtypes E and D (Gobbin et al. 2003), definitively split in lineages A4-B1 and A4-B2 by the use of ORF B information. Thus, five main divergent and four secondary viral lineages were detected within the CHV1 population from western Europe. Lineages A1-B1, A2-B1 and A3-B3 correspond, respectively, to the subtypes F1, F2 and I, which were previously defined by a RFLP approach and ORF A sequencing (Alleman et al., 1999; Gobbin et al. 2003).

Our finding of one fungal isolate infected by two different viral lineages provides evidence that such multiple infections in *C. parasitica* may occur in populations where different viral lineages coexist. Multiple CHV1 infections have previously been suspected in one *C. parasitica* isolate (Elliston 1985), but not genetically resolved. Mixed virus infections could have a synergistic effect on both viruses, as observed in a double infection by CHV1 and a MyRV1 a mycoreovirus (Sun et al. 2006) or a deleterious effect, as observed for a mixed infection by CHV3 (Smart and Fulbright 1995). When different strains of the same virus coinfect the same host, hybrid molecules can be produced during the replication process resulting in homologous recombination. In this study, we clearly identified recombination footprints in a majority of the viral strains (83%) within ORF A and ORF B, but also between the two ORFs. Two recombination events have resulted in two CHV1 lineages, which are widely spread and more frequent than the putative parental strains. The lineage A1-B1, comprised of subtype F1 strains, was likely derived through recombination between ancestral strains in the A4-B1 and A1-B2 lineages. This recombination event might have occurred in western France or in Spain where both parental lineages and the recombinant one are found. CHV1 viruses related to subtype F1 were also recently described in northern Spain indicating an expanded distribution range of this subtype (Zamora et al. 2012). The lineage A3-B3, corresponding to the widespread subtype I, could have resulted from a recombination event (E2) within ORF B involving strains from the A1-B2 lineage and, at least one, unknown strain. In contrast, the events E3 and E4 gave rise to two lineages detected with a low frequency. However, these recombination events might be more recent than the two previous ones, and the viruses may not have had enough time to spread efficiently. The emergence of hypovirulence observed in western and southern Europe associated with the spread of a few recombinant genotypes may have resulted from a beneficial effect of recombination events on CHV1 individuals. Experimental evidence has shown that recombination between two viral genomes (differing by 20%) has more often a beneficial than deleterious effect (Vuillaume et al. 2011). Random point mutations are often deleterious or lethal; however, homologous recombination creates new genotypes resulting from the integration of genomic regions into recipient genomes, both of which have already been under selective pressure. Such a beneficial effect can be compared to the positive effect that hybridization can have on the fitness of herbivorous insects used as biocontrol agents (Szucs et al. 2012) or to the effect of genetic recombination on the adaptive potential of introduced invasive species (Lavergne and Molofsky 2007; Keller and Taylor 2010).

However, recombination events make any inference about the origin and evolutionary trajectories of CHV1 lineages difficult. The size of the recombination blocks and breakpoint positions could not be determined without the sequence that lay between the two regions analyzed in our study (i.e., 4460 nt retrieved from the complete genome sequences of [Euro7] and [EP713]), and the inferred recombination scenarios (parental lineages, location and date of the recombination) must be considered with caution. To infer the evolutionary histories, we analyzed two genomic regions (A_sh_ and B_sh_) without significant evidence of recombination, although the possibility that undetected recombination events cannot be completely ruled out, especially for ORF B. The occurrence of ancient interlineage recombinants in sequence datasets might have biased the estimated age of the most recent common ancestor by pulling the estimated date deeper in the past (Awadalla 2003; Duffy and Holmes 2008; Lefeuvre et al. 2010). Using the relaxed evolutionary clock model, we inferred a rate of substitution per site per year of 2.5 × 10^−4^ for ORF A_sh_ and 1.7 × 10^−4^ for ORF B_sh_. These values are similar to those previously estimated with a relaxed clock model applied on ORF A sequences (Bryner et al. 2012) and in the range of substitution rates of other RNA viruses (Duffy et al. 2008). Our data confirmed that the first divergence of the different lineages had occurred prior to the transcontinental migration of *C. parasitica* (which did not occur before the end of the 19th century), similarly to what Gobbin et al. (2003) suggested by using a different phylogenetic model. However, due to large confidence intervals, it is difficult to date, with precision, the radiation of CHV1. Taking into account OFR A_sh_, the radiation of the dominant lineages might coincide with the introduction of *C. parasitica* into America and Europe (around 1900 and 1930, respectively).

CHV1 diversity appeared geographically structured, mirroring the distribution of *C. parasitica* diversity. CHV1 diversity was higher in the Pyrénées and central France regions where *C. parasitica* populations are genetically more diverse, and lower in southeastern France where vc type and genotype diversity of *C. parasitica* is also lower (Robin et al. 2000, 2009; Dutech et al. 2010). In Pyrénées and central France, up to seven CHV1 lineages were detected (among 22 viral strains in each case). A close association was observed between the viral lineages A2-B1 and A4-B1 and *C. parasitica* gene pools G2 and G3, respectively. Bayesian analyses yielded a radiation occurring in the 1940s for A2 and A4 clusters and a most probable origin in the central and Atlantic Pyrénées. Thus, all these results are in agreement with an introduction of these viral lineages in southern France or northern Spain directly from China or Japan, as it has been suggested for the *C. parasitica* gene pools G2 and G3 (Dutech et al. 2012). Imported Asian chestnut plants resistant to ink disease (Heiniger and Rigling 1994; Robin et al. 2009) might have introduced both *C. parasitica* and its hyperparasitic virus into this area. The lineage A1-B1 appeared similarly associated with the gene pool G2 mainly in central Pyrénées and central France and to G1 mainly in central France. One hypothesis which could be tested by using a more exhaustive sampling is that the lineage A1B1 migrated together with G2 and G3 into Dordogne (central France) and there came into contact with fungal isolates from the G1 gene pool (Dutech et al. 2010). Migration was likely accelerated by the use of the viral A1B1 lineage for biological control in France since 1970 (Robin et al. 2010). That could explain why this viral lineage was transmitted to another host gene pool. A rare transmission event was not required because horizontal virus transmission can occur between gene pools (Brusini and Robin 2013).

In southeastern France, the lineage A3-B3 was dominant, matching findings from a previous study where 78 of 79 viral strains from this area belonged to subtype I (Robin et al. 2010). The most likely origin of this lineage was southeastern France or Italy, where *C. parasitica* was officially first reported in Europe in 1938, right after the radiation times estimated for clusters A3 and B3 (1912 and 1920, respectively). These radiation times also coincide with the First World War, during which chestnut wood had been imported into Italy from North America (Grente 1981). The A3-B3 lineage was clearly associated with the G1 fungal lineage, which most likely has a Japanese origin and was introduced into Europe via North America (Dutech et al. 2012). These results are in agreement with a genetic analysis of ORF A with CHV1 strains, showing that Italian strains might have originated in Japan (Liu et al. 2007). All these data suggest that the viral A3-B3 lineage followed its host lineage G1 from Japan to Europe. However, the sticking point is that CHV1 did not establish in America (Milgroom and Cortesi 2004). Other *Cryphonectria* hypoviruses, never detected in Europe, are prevalent in North America but are typically much less virulent than CHV1 (Peever et al. 1997). The CHV1 lineage A3-B3 might have remained unnoticed in America, as a cryptic mycovirus, in the first years after the *C. parasitica* introduction, and then could have been vectored to Italy. Because the vc type diversity rapidly increased during the American chestnut blight epidemic, CHV1 could not persist. In Europe, this recombinant lineage adapted to its new environment and did spread and establish in the fungal founder population, which had a low vc type diversity. This scenario is supported by the predictions of an epidemiological model, which explains how a feedback mechanism exerted by the fungal host may result in selection against highly virulent viruses (Brusini et al. 2011). Another hypothesis is that the recombination event occurred in Europe after introductions of the ancestors in western France, followed by a quick and efficient spread of the recombinant strains in eastern France whereas the ancestors did not spread or disappear.

Both hypotheses suggest that the high prevalence of this recombinant lineage may be the result of adaptation and selection by the host and/or environment, which could also explain the close association between this viral lineage and the fungal lineage G1. Difference in fitness among CHV1 subtypes have been reported (Peever et al. 2000). A long-term field study also suggested that a greater ecological fitness of subtype I (lineage A3-B3) compared to subtypes F1 and F2 probably accounted for its greater invasiveness (Robin et al. 2010). Moreover, as fitness components can vary significantly depending on genotype × genotype × temperature interactions (Bryner and Rigling 2011), divergent selection could have favoured specific viral lineages in specific host populations and environments. We showed clear evidence that some amino acids in ORF A and ORF B were subject to positive selection, although such protein encoding regions are generally expected to evolve under conservative pressures (i.e., purifying selection). Interestingly, positive selection was detected only for the A3-B3 lineage, suggesting that host adaptation could have occurred.

Several recombination events and variable selection pressures contributed to CHV1 evolution, agreeing with a non-clock-like diversification rate. These two mechanisms may be at the origin of the CHV1 populations. Ancestors introduced in western France either gave raise to lineages which beneficiated to recombination and adapted to their new biotic and abiotic environment, or remained with low frequency in this area. Taking into account these two evolutionary processes is crucial for developing a biological control strategy against chestnut blight using CHV1. Our results provide evidence that divergent CHV1 lineages recombine and may adapt over time to establish in a new area. At this time, biocontrol with CHV1 relies on a method intermediate between a classical control method (using an exotic hyperparasite of an invasive pest species) and an augmentative method (increasing the density and incidence of an already naturally occurring enemy). Using multiple CHV1 lineages in areas where classical biological control is still attempted may be an option to promote beneficial recombinations between different lineages and new associations with hosts. However, the phenotypic and genetic diversity of CHV1 may also have an effect for *C. parasitica* management. Indeed, theoretical studies predict that virulence should be modified when multiple strains of parasites regularly compete within hosts (Franck 1996). When releases of a hyperparasite aim at a successful regulation of primary parasite populations, one can wonder whether strains with the highest virulence are the best ones to use. Morozov et al. showed that mild strains of the hyperparasite, characterized by a high vertical transmission rate and low virulence, more readily establish in a host population than severe strains (Morozov et al. 2007). On the contrary, in areas where CHV1 lineages are well established, our results suggest that one should use adapted lineages, which naturally spread and which could out-compete non-adapted ones.

Classical biological control relies on deliberate biological invasions and hence has been used to study the evolution of microorganisms introduced into new areas and how their invasion success may depend on demographic, genetic and environmental factors (Hufbauer and Roderick 2005; Roderick et al. 2012; Fauvergue et al. 2012). Vice-versa, incorporating evolutionary principles in agriculture, as advocated by Hendry et al. (2011), would be especially beneficial to control diseases with an environmentally friendly approach. Thus, insights gained in the scientific field of invasion biology should also be used to improve applied biological control. Genetic recombination and selection events may explain the establishment success of invasive organisms (Facon et al. 2008). This evolutionary process may be enhanced if invasive species are originating from different gene pools or hybridize with local genotypes or species (for example Lavergne and Molofsky 2007; Keller and Taylor 2010). These lessons and those learned from the vigor of hybrids (Szucs et al. 2012) and high recombination rates in viruses (Vuillaume et al. 2011; Cory and Franklin 2012) could open new perspectives for classical biological control. For the chestnut blight disease for example, diversifying the origins of CHV1 might be a solution in northern America where repeated failures of establishment of one specific genotype have been reported.

## Data archiving

CHV1 sequences were deposited in the NCBI data base (Genbank accession numbers JF790918.1 to JF790965.1, JF795811.1 to JF795863.1, and JG778845.1 to JG778852.1).

## Supporting Information

**Figure S1.** Nucleotide frequency and phylogenetic signal saturation analyses for the ORF-A and ORF-B genes of CHV1.

**Figure S2.** Strict consensus from trees resulting from the maximum-parsimony analyses (MP) of *Cryphonectria hypovirus 1* ORF A and ORF B sequences.

**Figure S3.** Lineage divergence dates inferred from concatenated *Cryphonectria hypovirus 1* sequences.

**Figure S4.** Maximum clade credibility trees constructed from the ORF A_sh_ and ORF B_sh_ *Cryphonectria hypovirus 1* datasets.

**Table S1.** List of recombination events (E1-5) detected in *Cryphonectria hypovirus 1* the recombination detection methods implemented by the program RDP3.

**Table S2.** Bayes Factors [log10(BF)] calculated from the marginal likelihoods of three competing population genetics models [Constant size (CS), Exponential (Expo), Expansion (Expan)] and using Tracer 1.4: analyses were conducted on subsets of ORF A ORF B alignments (ORF Ash and ORF Bsh) including only non-recombinant sequence blocks to avoid recombination bias.

**Table S3**. Estimation of substitution rates in *Cryphonectria hypovirus 1* according to different population genetic models and on subsets of ORF A and ORF B alignments (ORF Ash and ORF Bsh).

**Data S1.** Selection of the best-fitting model.

**Data S2.** Positive selection analyses with CODEML.
