# Supplementary material for: Multiple introductions and recombination in Cryphonectria hypovirus 1: perspective for a sustainable biological control of chestnut blight
Source: Evol Appl. 2014 Apr 15;7(5):580–96. doi: 10.1111/eva.12157 (PMC4055179; doi:10.1111/eva.12157)
Supplement: Supplementary file 1 [file eva0007-0580-SD1.pptx]

## Slide 1
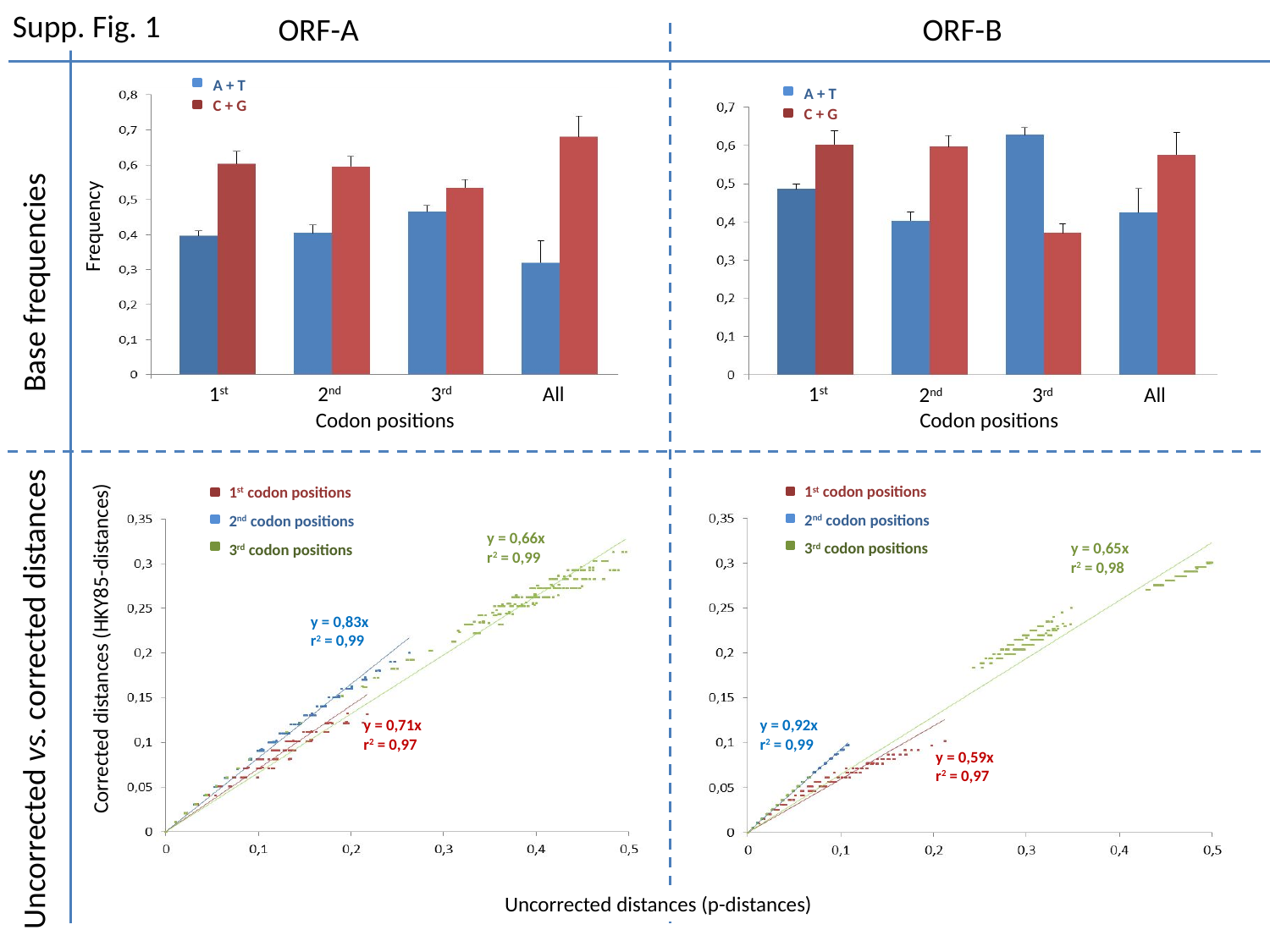

Supp. Fig. 1
ORF-A
ORF-B
A + T
C + G
A + T
C + G
Base frequencies
Frequency
1st
3rd
1st
2nd
All
3rd
2nd
All
Codon positions
Codon positions
Corrected distances (HKY85-distances)
Uncorrected vs. corrected distances
1st codon positions
1st codon positions
y = 0,65x
r2 = 0,98
y = 0,92x
r2 = 0,99
y = 0,59x
r2 = 0,97
2nd codon positions
2nd codon positions
y = 0,66x
r2 = 0,99
3rd codon positions
3rd codon positions
y = 0,83x
r2 = 0,99
y = 0,71x
r2 = 0,97
Uncorrected distances (p-distances)

## Slide 2
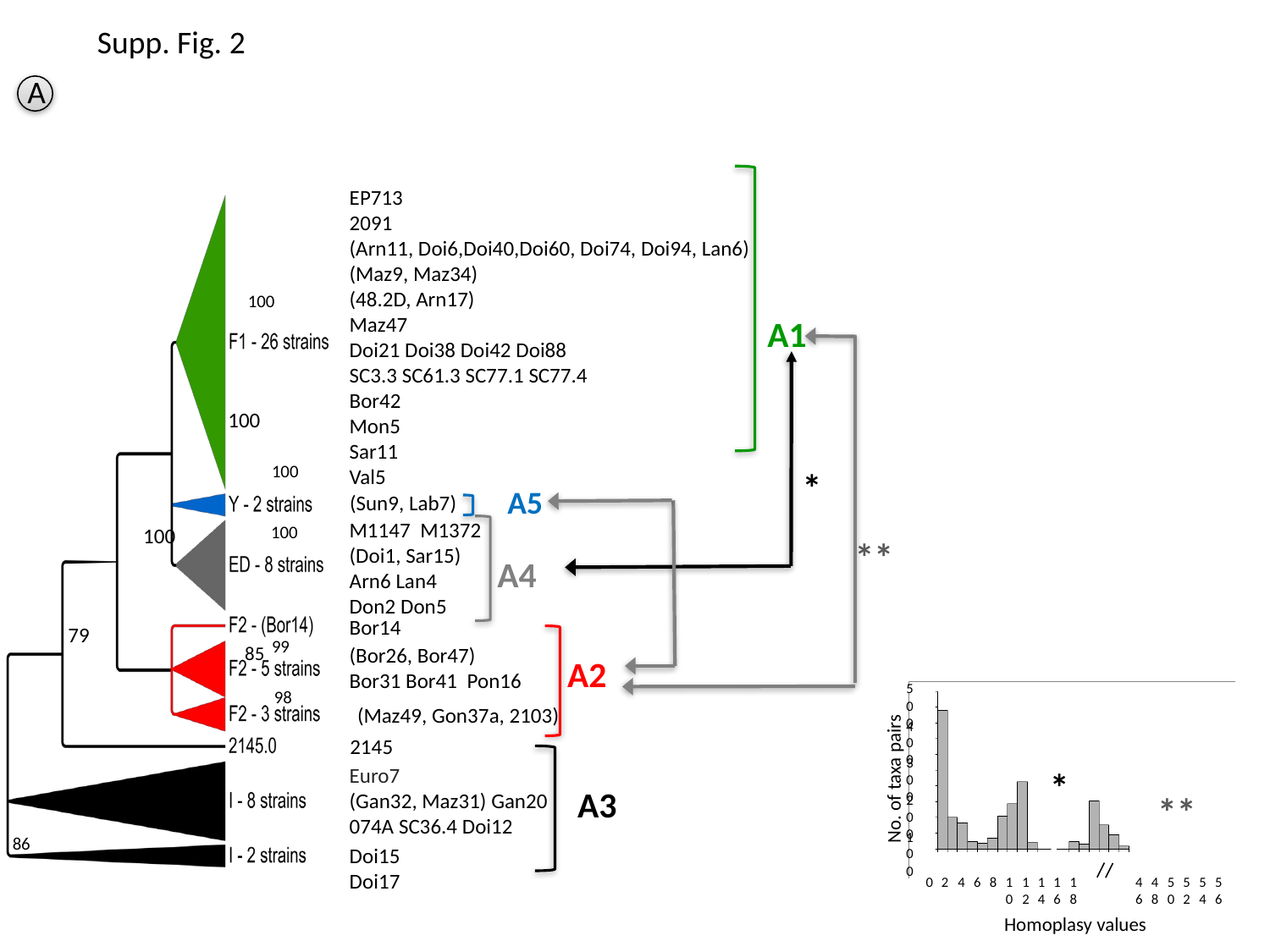

Supp. Fig. 2
A
EP713
2091
(Arn11, Doi6,Doi40,Doi60, Doi74, Doi94, Lan6)
(Maz9, Maz34)
(48.2D, Arn17)
Maz47
Doi21 Doi38 Doi42 Doi88
SC3.3 SC61.3 SC77.1 SC77.4
Bor42
Mon5
Sar11
Val5
100
A1
**
*
100
100
A5
(Sun9, Lab7)
M1147 M1372
(Doi1, Sar15)
Arn6 Lan4
Don2 Don5
100
100
A4
Bor14
79
99
85
(Bor26, Bor47)
Bor31 Bor41 Pon16
A2
500
400
300
*
No. of taxa pairs
**
200
100
0
2
4
6
8
10
12
14
16
18
46
48
50
52
54
56
Homoplasy values
98
(Maz49, Gon37a, 2103)
2145
Euro7
(Gan32, Maz31) Gan20 074A SC36.4 Doi12
A3
86
Doi15 Doi17

## Slide 3
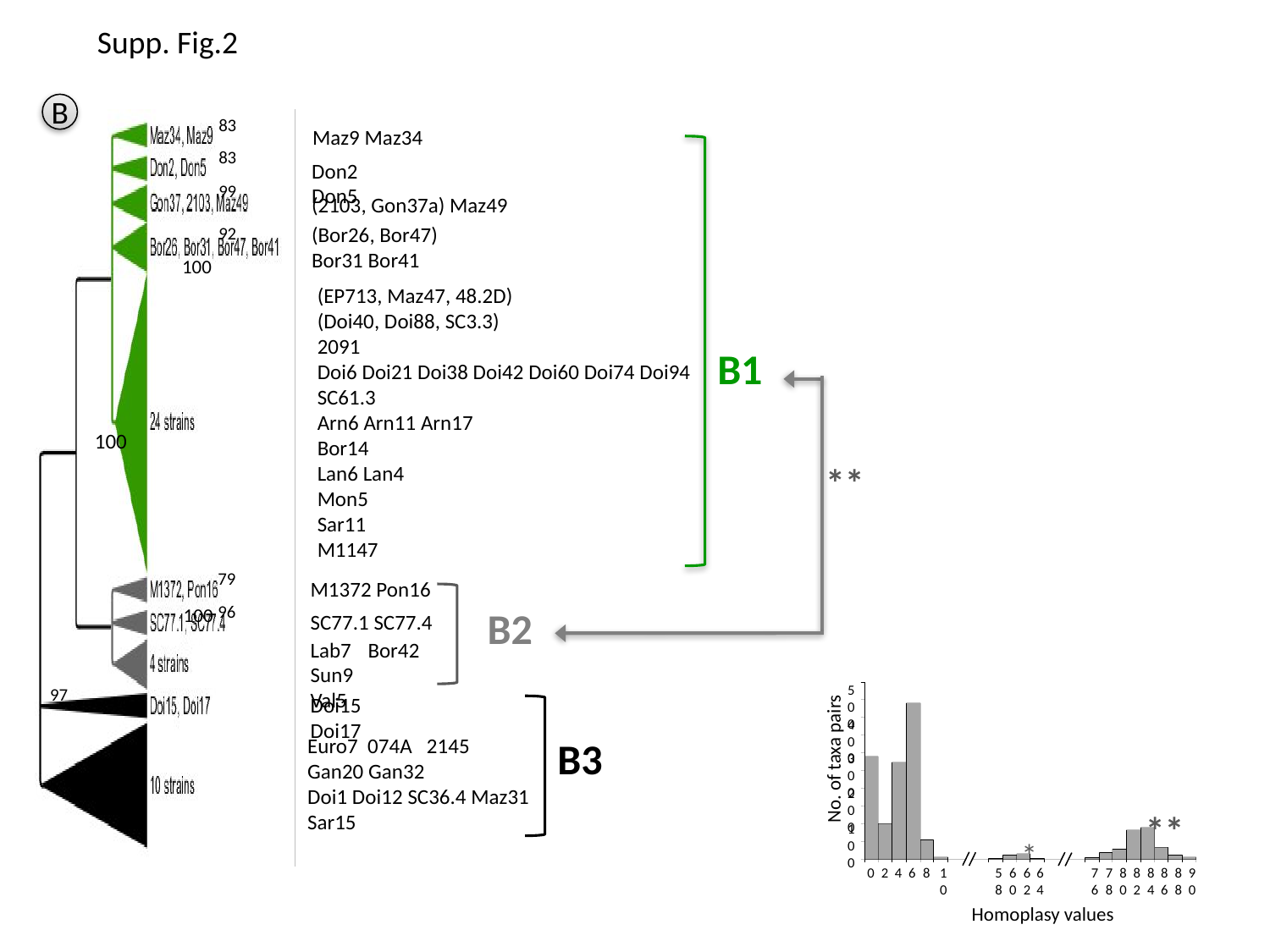

Supp. Fig.2
B
83
Maz9 Maz34
83
Don2 Don5
99
(2103, Gon37a) Maz49
(Bor26, Bor47)
Bor31 Bor41
92
100
(EP713, Maz47, 48.2D)
(Doi40, Doi88, SC3.3)
2091
Doi6 Doi21 Doi38 Doi42 Doi60 Doi74 Doi94
SC61.3
Arn6 Arn11 Arn17
Bor14
Lan6 Lan4
Mon5
Sar11
M1147
B1
**
100
79
M1372 Pon16
96
B2
100
SC77.1 SC77.4
Lab7 Sun9 Val5 Bor42
500
400
300
No. of taxa pairs
200
**
100
*
88
90
0
2
4
6
8
10
58
60
62
64
76
78
80
82
84
86
Homoplasy values
97
Doi15 Doi17
Euro7 074A 2145
Gan20 Gan32
Doi1 Doi12 SC36.4 Maz31
Sar15
B3

## Slide 4
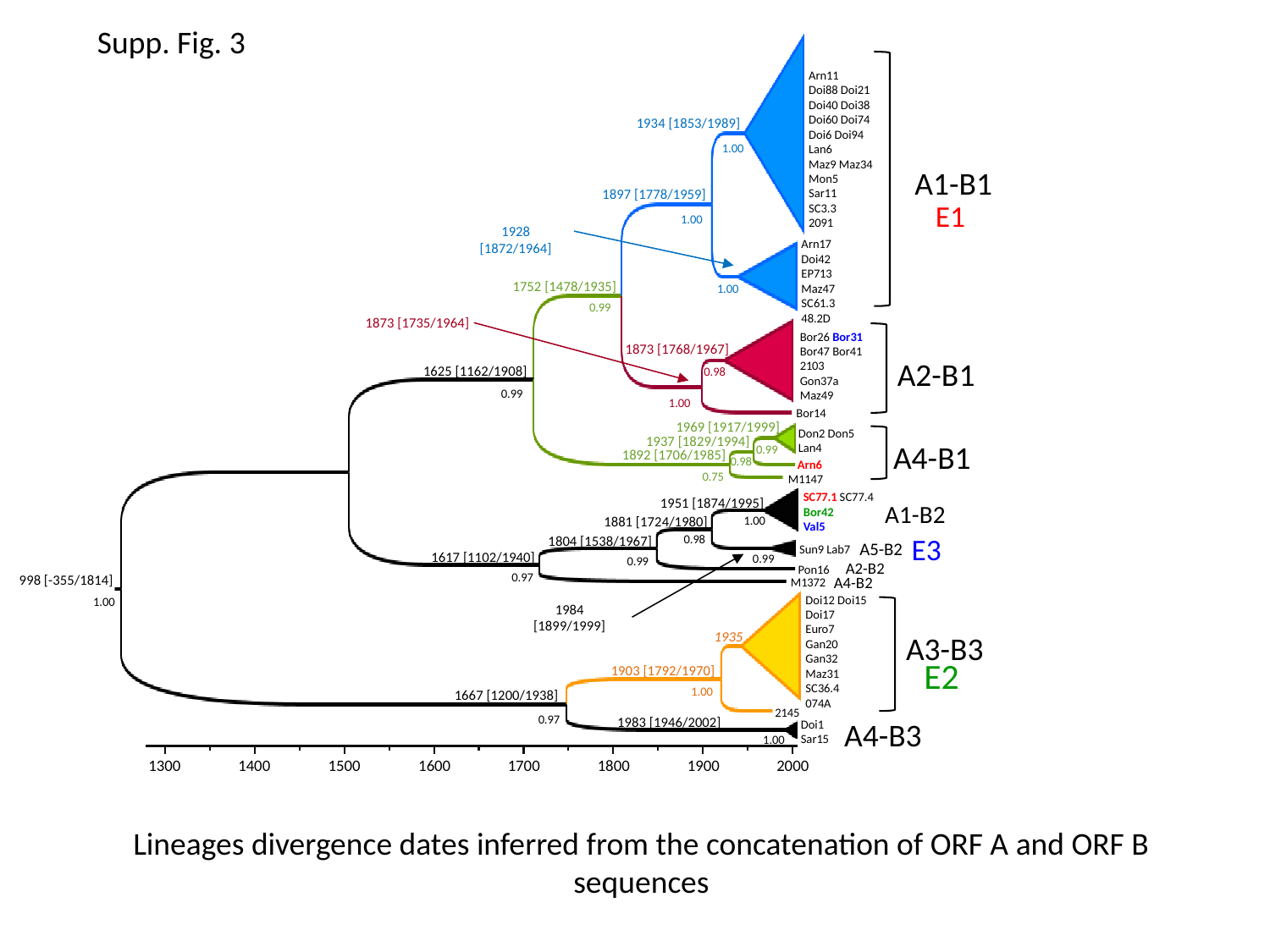

Supp. Fig. 3
Arn11
Doi88 Doi21
Doi40 Doi38
Doi60 Doi74
Doi6 Doi94
Lan6
Maz9 Maz34
Mon5
Sar11
SC3.3
2091
1934 [1853/1989]
1.00
A1-B1
1897 [1778/1959]
E1
1.00
1928 [1872/1964]
Arn17
Doi42
EP713
Maz47
SC61.3
48.2D
1752 [1478/1935]
1.00
0.99
1873 [1735/1964]
Bor26 Bor31
Bor47 Bor41
2103
Gon37a
Maz49
1873 [1768/1967]
A2-B1
1625 [1162/1908]
0.98
0.99
1.00
Bor14
1969 [1917/1999]
Don2 Don5
Lan4
1937 [1829/1994]
A4-B1
0.99
1892 [1706/1985]
0.98
Arn6
0.75
M1147
SC77.1 SC77.4
Bor42
Val5
1951 [1874/1995]
A1-B2
1.00
1881 [1724/1980]
E3
0.98
1804 [1538/1967]
A5-B2
Sun9 Lab7
1617 [1102/1940]
0.99
0.99
A2-B2
Pon16
0.97
998 [-355/1814]
A4-B2
M1372
Doi12 Doi15 Doi17
Euro7
Gan20 Gan32
Maz31
SC36.4
074A
1.00
1984 [1899/1999]
1935
A3-B3
E2
1903 [1792/1970]
1.00
1667 [1200/1938]
2145
0.97
1983 [1946/2002]
A4-B3
Doi1
Sar15
1.00
1600
1700
1800
1900
1300
2000
1400
1500
Lineages divergence dates inferred from the concatenation of ORF A and ORF B sequences

## Slide 5
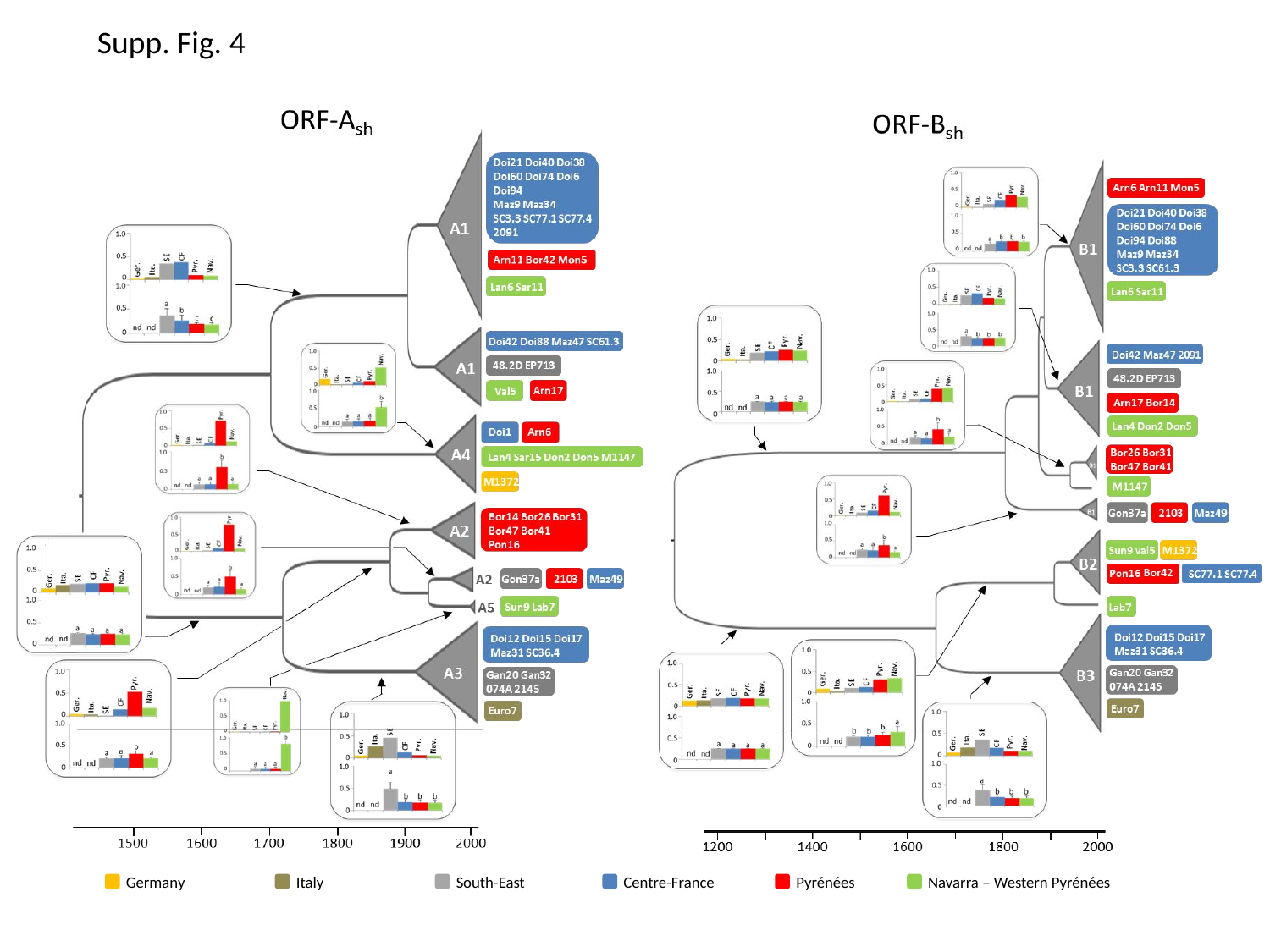

Supp. Fig. 4
Germany
Italy
South-East
Centre-France
Pyrénées
Navarra – Western Pyrénées
