# Supplementary material for: Multiple introductions and recombination in Cryphonectria hypovirus 1: perspective for a sustainable biological control of chestnut blight
Source: Evol Appl. 2014 Apr 15;7(5):580–96. doi: 10.1111/eva.12157 (PMC4055179; doi:10.1111/eva.12157)
Supplement: Supplementary file 2 [file eva0007-0580-SD2.pdf]

Table S1. List of recombination events (E1-5) detected in *Cryphonectria hypovirus* 1 the recombination detection methods implemented by the program RDP3.

Event Number	Breakpoint Positions		Sequences			Detection Methods						
	begin	end	Recombinant Sequence(s)	Minor Parental	Major Parental	RDP	GENECONV	Bootscan	Maxchi	Chimaera	SiSscan	3seq
**E1**	1	309	EP713, 2091, 48.2D, Arn11,-17, Doi6, -21, -38, -40, -42, -60, -74, -88,- 94, Lan6, Maz9, -64, -47, Mon5, Sar11, SC3.3, -61.3	SC77.1	Arn6	2.275×10^{-06}	1.600×10^{-06}	1.416×10^{-08}	9.127×10^{-09}	3.139×10^{-09}	1.036×10^{-09}	5.234×10^{-16}
**E2**	707	899	Euro7, Doi1, -12, -15, -17, Gan20, -32, 2145, 07.4A, Maz31, Sar15, Sc36.4	unknown	Bor42	2.000×10^{-05}	2.814×10^{-04}	3.144×10^{-06}	2.370×10^{-03}	1.554×10^{-02}	9.168×10^{-10}	7.267×10^{-04}
**E3**	199	739	Lab7, Sun9, Pon16	Val5	Bor31	1.006×10^{-04}	4.335×10^{-06}	1.013×10^{-04}	8.241×10^{-07}	1.222×10^{-06}	4.200×10^{-16}	5.234×10^{-16}
**E4**	303	-889	M1372, Doi1, Sar15	Don2	Maz31	9.329×10^{-04}	2.798×10^{-08}	5.162×10^{-10}	2.583×10^{-11}	8.396×10^{-11}	3.375×10^{-16}	3.375×10^{-16}
**E5**	204	-323	Gon37a, Bor14, -26, -31, -	unknown	Lab7			2.363×10^{-02}	4.256×10^{-04}		1.977×10^{-05}	3.203×10^{-06}

2987	3444	AF509739		4.50E-35	2.39E-17	6.13E-25	9.53E-12	8.23E-14	3.48E-10
		AJ586885							
		AF104036							
		AF326775							
		AJ132548							
		AJ579307							
		AJ314737							

3616	2509	AF422174		1.10E-09	4.51E-43	4.87E-03	1.46E-12	4.48E-03	NS
3246*	3401	AJ579307<br>AJ314737[P]		5.72E-03	NS	NS	NS	6.69E-05	NS

18*	2808	AY090558	9.20E-29	3.02E-39	2.04E-24	3.03E-19	7.17E-03	NS

1617	2007	AF326775	4.78E-24	2.07E-28	8.13E-08	1.50E-14	3.04E-18	7.87E-26
3075	3184	NDNV<br>M88686	5.19E-09	2.89E-25	1.02E-17	9.32E-07	1.95E-06	1.34E-12

18*	2855	AF039031	3.26E-09	5.48E-24	6.19E-10	3.46E-13	1.10E-08	NS

664	1579	X84735<br>AF029217<br>AY190290<br>AB085793<br>AF509739<br>U15015<br>Y16421<br>AF509743<br>U15016<br>AF102276<br>AY184487<br>AY514631<br>AF206674<br>U88692<br>AJ006459<br>Z83257[P]<br>AF112354[P]<br>AJ422132[P]<br>AF422174<br>AJ314737[P]<br>J02057	2.48E-36	1.94E-30	4.14E-30	6.60E-16	2.27E-26	NS

5*	2566	AJ717569<br>AJ422132<br>AF155806	2.69E-21	1.30E-33	5.88E-25	1.39E-23	1.20E-19	NS
2859	3616*	AJ508784<br>AF130415	5.66E-15	2.55E-26	1.16E-14	1.42E-11	6.49E-13	NS

733	1684	AF130415	8.13E-21	2.06E-17	1.40E-17	1.83E-22	8.34E-19	5.87E-13
2864	3030*	D00940<br>K02029<br>NDNV	9.64E-22	2.14E-09	3.38E-15	3.60E-11	1.49E-11	6.73E-06

		DQ336350						
		AJ557450						
		AJ557451						
		ToMLCV7						
		AY090555						
		M88686						
		AY120882						
		AY339619						
		AY339618[P]						
		AY029750						
		AF291705						
3069	3371	AY727903	1.21E-20	1.22E-12	2.02E-16	1.44E-12	1.71E-09	NS
		AF126406						
		AJ627904						
		E00957						

2869	3283	Y15034<br>AF490004<br>AY090557<br>ToMoLCV9<br>EF417915	7.37E-19	8.28E-08	5.86E-15	1.66E-14	5.34E-15	3.69E-15

2644	2860	AY514632		1.38E-18	1.43E-03	1.19E-11	3.32E-07	1.88E-08	1.50E-06
3375*	3569*	DQ178608		1.46E-06	6.53E-18	6.10E-14	4.01E-09	5.23E-04	7.57E-17
3087	3401	AJ968370<br>AF326775<br>X97203<br>AY548948<br>AY134867		1.05E-18	1.05186E-07	3.81E-10	2.55E-12	8.61E-10	6.18E-20

1616*	1734	AY134867		1.45E-11	6.90E-18	5.42E-10	4.81E-04	NS	4.64E-14
2904	3061*	M24597		1.59E-16	3.37E-05	6.80E-13	NS	NS	1.86E-05

2917	3140	AB085793	4.44E-11	NS	5.53E-08	7.50E-04	3.86E-04	NS
3374	3534*	AF139168	1.02E-09	1.67E-09	1.26E-08	2.09E-04	2.34E-03	3.10E-04

2864*	3150	AF314145	2.58E-09	0.025168578	2.33E-03	8.04E-04	NS	NS
3371*	3511*	AB085793	4.78E-09	NS	1.72E-05	1.79E-07	2.60E-04	NS
3605	25	AY508993<br>K02029<br>NDNV<br>DQ336350<br>AJ557450<br>AJ557451<br>ToMLCV7<br>AY090558[P]<br>AY090555<br>AF101476<br>AF239671	1.98E-05	1.13E-19	1.15E-09	9.81E-08	3.06E-05	NS

X99550
X70418
M88179
Y11099
L01635
AY044133
U51137
AF049336
AJ608286
AY044135
AJ344452
M88686
NC_004659
DQ347945
DQ022611
AY083351
AY742220
Y14874
U77964
AF012300
L14460
Y15034
D00940
AY120882
AY339619
AY339618
AF068636
AY508991
DQ178608[P]
U65529[P]
DQ406672
AY029750
AF490004

		AY090557							
		AF291705[P]							
		U57457							
		AF149227[P]							
		AF224760							
		AF325497							
		AY064391[P]							
		M38183							
		AF421552							
		EuMV							
		AF110189							
		ToMoLCV9							
		EF417915							
662	1514	AY727903		9.75E-18	2.55E-17	8.49E-19	2.39E-08	7.54E-15	NS

3287	3519	AY339618	2.94E-17	NS	4.72E-18	4.17E-05	1.52E-02	7.16E-06
3610	914	DQ347950<br>K02029<br>AF239671<br>AJ549960<br>U51137<br>AY044135<br>M38183<br>AF132852:2B	6.60E-17	1.68E-12	1.12E-08	8.84E-10	3.66E-13	NS
964	1541	M24597<br>X97203	8.25E-17	4.21E-02	1.54E-09	5.89E-06	1.43E-09	NS

2953	3401	AF261885<br>AB085793<br>AJ575819[P]	1.01E-17	1.00117E-06	7.42E-10	2.73E-11	1.83E-08	4.23E-12

28*	854	AY795983	1.05E-17	6.78E-03	2.84E-05	7.90E-14	8.07E-12	NS
		AY514632						
		AJ495812[P]						
		X63015						
		AJ810096						
		AJ006459[P]						
		AJ006458						
		AF112354[P]						
		Z83256						
		AJ579307						
		AF261885						

3042	3352	AY514631<br>AY190290<br>AY514632<br>AJ495812<br>AF206674<br>X63015<br>U88692<br>AB055009	1.37E-15	NS	4.52E-13	9.53E-10	3.29E-10	NS

836	963*	M24597		1.01E-18	1.56E-05	1.06E-07	NS	NS	NS

2871	3533*	AF239671<br>X70418[P]<br>DQ347950	1.41E-03	NS	NS	1.47E-11	5.43E-05	NS
3065	3401	AJ810096<br>AF422174<br>AF511529	3.13E-09	NS	1.02E-05	2.14E-07	8.66E-09	3.39E-09

2859	2962	X99550<br>M88179<br>AJ608286<br>NC_004659<br>DQ022611<br>Y14874[T]	3.41E-09	8.24E-05	1.86E-03	4.70E-03	1.29E-03	2.55E-02
1575*	1992	AF239671<br>X70418<br>DQ347950<br>AY044133	2.11E-04	4.25E-08	2.00E-02	3.97E-09	2.02E-07	NS
2629	2978*	AF509739	1.01E-14	NS	3.03E-14	8.31E-10	2.95E-03	3.35E-09

3402*	3519	AJ717569<br>AJ422132<br>AF155806	3.06E-14	9.10E-13	8.81E-11	3.90E-10	8.09E-05	4.35E-19
3112	3377	U15015<br>Y16421<br>AF509743<br>U15016<br>AF102276<br>AY184487<br>U88692[P]<br>Z24758	1.02E-13	3.12E-02	2.09E-11	3.62E-11	3.60E-12	2.24E-07

2857	3028*	E00957<br>AF126406<br>AJ627904	2.32E-13	5.85E-09	1.12E-09	1.20E-03	4.38E-07	3.53E-11
2149	2857*	AY044133<br>X70418<br>AY727903	1.03E-15	4.28E-02	6.29E-14	1.92E-08	1.94E-12	NS

3284*	3569*	ToMoLCV9	5.46E-13	2.56E-13	1.33E-11	7.42E-09	6.55E-08	NS
3376	3569*	U77964<br>AY090557	7.73E-13	7.78E-05	2.08E-11	3.00E-09	2.49E-08	1.25E-10

1846	2385	AJ586885<br>AF104036<br>AJ132548		1.23E-12	NS	4.64E-04	3.29E-07	1.09E-05	NS

661	1513	AY134867	1.50E-35	3.56E-28	7.58E-23	4.58E-10	7.16E-22	NS

470	588*	AF422174<br>Z83257<br>AJ717569	1.92E-14	2.86E-05	2.26E-04	7.10E-07	6.25E-08	NS
2427	2499	AJ557451<br>Y11099<br>NC_004659<br>DQ347945<br>AY083351<br>AY120882[T]	1.26E-12	NS	4.06E-10	NS	0.0218431	3.56E-06

3355	3533*	L14460<br>AF101476<br>M88179<br>Y11099<br>U51137[P]<br>AF049336<br>NC_004659<br>DQ347945<br>DQ022611<br>AF490004	1.80E-12	1.41E-02	2.96E-08	1.33E-04	3.68E-06	1.82E-10
1120	1499	AF126406<br>AJ968370[P]<br>AF314145<br>AJ627904<br>E00957	2.47E-08	NS	7.23E-04	2.65E-12	9.92E-11	2.87E-04

2767	3028*	AB055009	5.00E-12	4.55E-08	5.53E-09	3.00E-02	2.09E-03	NS
799	1287	AF326775<br>AJ586885<br>AF104036<br>AJ132548	6.90E-14	NS	3.06E-06	2.23E-12	3.06E-06	NS

3458*	3617*	M24597	2.63E-06	7.20E-21	1.52E-04	4.58E-13	4.43E-03	1.28E-02
3445*	3551*	AF326775<br>AJ586885<br>AF104036<br>AJ132548<br>AF511529	3.65E-11	5.32E-06	1.41E-06	1.90E-11	4.24E-07	NS
3355	3536*	D00940<br>AF012300<br>AY120882	3.43E-11	1.82E-03	2.46E-10	1.38E-04	3.92E-04	5.72E-15
3109	3368*	J02057	4.82E-11	NS	1.86E-11	7.79E-05	3.37E-03	4.29E-06

328	652*	U49907	1.29E-03	1.63E-17	2.21E-03	5.53E-11	3.94E-04	NS
3080*	3277*	AY339619	6.50E-11	NS	1.66E-09	1.59E-06	NS	NS

663	872	AB055009<br>Y16421[T]<br>U88692<br>Z24758[T]	8.64E-05	1.89E-09	7.77E-04	3.08E-08	2.43E-04	NS

1935	2008	X84735<br>AF029217[T]<br>M24597[T]<br>X97203<br>AY548948<br>AY134867	2.34E-06	NS	4.21E-02	2.23E-03	NS	NS
662*	750	AF509743<br>AB085793<br>AF509739<br>U15015<br>U15016<br>AF102276<br>AY184487<br>AF155806<br>J02057	6.05E-06	1.29E-02	NS	1.47E-03	3.19E-04	NS

334*	589*	AY184487	1.01E-05	1.08E-02	2.12E-05	1.51E-02	1.46E-03	1.81E-06
1642	1768	AB055009	5.73E-08	NS	4.05E-03	3.83E-02	9.37E-04	NS

663*	875	AY190290<br>AJ314737	1.55E-09	8.41E-03	4.76E-03	4.50E-11	1.05E-08	NS

2864	3278	AF139168	NS	NS	2.76E-03	2.34E-10	NS	NS

1635	1725*	AY548948	3.07E-10	NS	1.25E-03	8.90E-09	1.36E-02	NS

966	1519	AY339619<br>AJ508784<br>AY339618	2.42E-09	NS	8.46E-08	3.29E-10	5.80E-07	1.52E-09
3448*	3611*	AF509739	4.05E-04	1.04E-10	4.92E-03	3.41E-10	2.44E-02	NS

689	1539	AJ608286	1.31E-02	NS	4.34E-02	4.02E-10	2.51E-08	NS
2784	2899*	M24597	5.04E-10	NS	5.04E-09	3.52E-03	0.03188224	NS

2873*	3027	DQ178608	2.40E-02	NS	NS	7.02E-10	5.06E-06	2.56E-08
663	934*	AJ968370<br>AY514632<br>AF511529<br>AF126406	NS	3.07E-05	2.03E-04	2.06E-05	NS	NS
2478	2677	U49907	8.21E-05	NS	1.86E-02	NS	NS	NS

		M24597[T]<br>AY548948[T]<br>AY134867[T]						
3065*	3369	M24597	1.08E-04	2.03E-07	4.00E-06	2.85E-04	6.54E-04	6.33E-07
1360	2297	AF139168<br>AJ549960	NS	NS	2.17E-05	3.51E-04	4.42E-05	8.65E-05
2786*	3033*	AY190290<br>U88692	1.15E-04	5.37E-12	2.04E-09	6.44E-03	NS	NS
915*	2066	AF239671<br>DQ347950	9.91E-04	NS	5.90E-03	2.21E-06	2.13E-06	NS
2372	2622	AJ012081	8.34E-04	NS	2.65E-03	1.31E-03	NS	NS

916	1011	AF261885<br>AJ006458[T]<br>AY795983[T]<br>Z83256[T]	1.56E-03	NS	5.65E-03	NS	NS	NS
3364	3531*	AJ557451	1.13E-03	9.20E-05	3.14E-03	7.24E-03	7.45E-03	NS
3490*	3521	AY514632<br>AY514631[T]<br>AF206674[T]	1.73E-03	NS	0.029915344	NS	NS	NS
2987	3036	ToMoLCV9<br>AF490004[T]<br>AY090557[T]	2.28E-03	4.77E-02	1.80E-03	NS	NS	NS
3297	3394*	AF509739	2.19E-02	NS	NS	2.50E-03	2.58E-02	NS
2561	2858*	AF110189	1.91E-07	NS	NS	3.98E-03	NS	NS
1279	1637	AY044135	4.34E-02	NS	2.09E-03	5.32E-03	3.75E-02	NS
2808	2920	AF326775	6.01E-03	NS	5.34E-04	NS	NS	NS

3605	17*	AY727903	NS	5.03E-04	2.97E-02	NS	NS	NS
1992	2145	AY508991	4.52E-03	NS	4.79E-03	1.26E-02	NS	NS
3175	3232	X84735<br>AY044133	6.08E-03	NS	1.12E-02	NS	NS	NS
3042*	3365*	AY090555<br>DQ336350[T]<br>AJ557450	8.24E-03	1.78E-02	4.47E-03	NS	NS	0.028984179
1389*	1579	AJ586885<br>AF104036[T]<br>AF326775[T]<br>AJ132548[T]	8.53E-03	NS	4.21E-02	NS	NS	NS
2767	2857*	AJ968370<br>AJ586885<br>AJ012081[T]<br>Z24758[T]<br>AJ006458<br>Z83256<br>AF261885<br>J02057[T]<br>AF511529[T]<br>AF314145<br>AF126406<br>AJ627904	9.71E-09	9.10E-03	5.27E-04	2.92E-02	2.04E-02	NS

		E00957						
2745	2864*	U49907	3.13E-06	2.64E-04	8.68E-03	8.92E-03	NS	4.22E-07

3305*	3346	AY190290<br>AJ012081[T]<br>AJ006459[T]<br>AJ006458[T]<br>AY795983[T]<br>Z83257[T]<br>AF112354[T]<br>Z83256[T]<br>AJ717569[T]<br>AJ557451[T]<br>ToMLCV7[T]	6.92E-05	NS	0.036618931	NS	NS	NS
2970*	3289	AY064391<br>AF029217[T]<br>AY742220[T]<br>U65529<br>DQ406672[T]<br>AF149227<br>AF224760<br>AF325497<br>EuMV[T]<br>U49907[T]	8.10E-14	3.12E-09	1.73E-09	5.23E-05	5.72E-03	NS
3269	3357*	AY508991<br>AJ549960[T]<br>AJ344452[T]	3.50E-07	NS	NS	4.67E-04	NS	NS

		AF068636[T]						
2299*	2344	DQ022611<br>X99550[T]<br>AY044135[T]<br>DQ347945	1.45E-03	NS	1.14E-03	NS	NS	NS
2978	3101*	J02057<br>AF029217[T]<br>U15015[T]<br>AF509743[T]<br>AY184487[T]<br>AY514631[T]<br>AF206674[T]<br>X63015[T]	2.12E-03	NS	7.65E-03	NS	NS	NS
2942*	3070*	AY548948	3.62E-03	NS	8.76E-03	0.01981719	NS	6.23E-10
380	470	D00940<br>Y14874[T]<br>Y15034[T]<br>AY120882[T]<br>AF490004[T]<br>AY090557[T]<br>AF291705[T]	6.70E-03	2.33944E-05	NS	NS	NS	NS
1836	1873	AJ314737<br>AY190290[T]<br>AF509739[T]<br>AF509743[T]<br>AY184487[T]<br>AY514631[T]	8.23E-03	NS	4.88E-02	NS	NS	NS

		AJ495812[T]<br>Z24758[T]<br>AJ006459[T]<br>AJ006458[T]<br>AY795983[T]<br>AF112354[T]<br>AJ422132[T]<br>AF422174[T]<br>AJ575819[T]<br>AJ579307[T]<br>J02057[T]						
3608	29*	U49907	8.93E-03	NS	4.30E-03	NS	NS	NS
3201	3286*	X97203<br>AF132852:2B[T]	1.63E-02	NS	1.14E-02	0.01093862	NS	NS
3131	3160*	AF126406	1.17E-02	NS	3.83E-02	NS	NS	NS
3453	22*	J02057	1.83E-02	NS	NS	NS	6.57E-03	NS
21*	270*	AY548948	1.26E-23	2.05E-02	4.81E-03	7.21E-07	1.78E-03	NS
2031*	2180	AF102276<br>AB085793[T]<br>AF509739[T]<br>U15015[T]<br>Y16421[T]<br>U15016[T]	2.20E-02	NS	4.33E-02	NS	NS	1.45E-07
2912*	3113	U49907	8.73E-29	2.74E-11	5.58E-22	2.24E-02	2.28E-05	NS

688	782*	AY064391<br>AF239671[T]<br>DQ347950[T]<br>AF068636[T]<br>DQ178608[T]<br>U65529[T]<br>U57457[T]<br>AF149227[T]<br>AF224760[T]<br>AF325497[T]<br>M38183[T]<br>AF421552[T]<br>EuMV[T]<br>AF110189[T]	2.25E-02	NS	8.50E-03	NS	NS	NS
3374*	3547*	AB055009	2.60E-02	NS	NS	NS	NS	1.69E-04
2820	2869*	Y14874<br>AF049336[T]<br>AJ608286[T]<br>DQ022611[T]<br>L14460[T]	3.11E-02	3.50E-08	NS	NS	NS	NS
