# Supplementary material for: Multiple introductions and recombination in Cryphonectria hypovirus 1: perspective for a sustainable biological control of chestnut blight
Source: Evol Appl. 2014 Apr 15;7(5):580–96. doi: 10.1111/eva.12157 (PMC4055179; doi:10.1111/eva.12157)
Supplement: Supplementary file 3 [file eva0007-0580-SD3.pdf]

Table S2: Bayes Factors (log10(BF)) calculated from the marginal likelihoods of three competing population genetics models (Constant size ("CS"), Exponential ("Expo"), Expansion ("Expan")) and using Tracer 1.4: analyses were conducted on subsets of ORF A ORF B alignments (ORF Ash and ORF Bsh) including only non-recombinant sequence blocks to avoid recombination bias.

ORF Alignment			lnP (model | data)	Strict			Lognormal			Expo		
				CS	Expo	Expan	CS	Expo	Expan	CS	Expo	Expan
A_{sh}	Strict	CS	-785.193	-	0.262	0.18	-0.376	0.182	0.351	-1.335	-0.714	-0.844
		Expo	-785.797	-0.262	-	-0.083	-0.639	-0.08	0.089	-1.598	-0.977	-1.107
		Expan	-785.606	-0.18	0.083	-	-0.556	0.003	0.172	-1.515	-0.894	-1.024
	Lognormal	CS	-784.327	0.376	0.639	0.556	-	0.558	0.727	-0.959	-0.338	-0.468
		Expo	-785.613	-0.182	0.08	-0.003	-0.558	-	0.169	-1.518	-0.897	-1.027
		Expan	-786.001	-0.351	-0.089	-0.172	-0.727	-0.169	-	-1.686	-1.065	-1.196
	Expo	CS	-782.118	1.335	1.598	1.515	0.959	1.518	1.686	-	0.621	0.491
		Expo	-783.548	0.714	0.977	0.894	0.338	0.897	1.065	-0.621	-	-0.13
		Expan	-783.249	0.844	1.107	1.024	0.468	1.027	1.196	-0.491	0.13	-
B_{sh}	Strict	CS	-1450.346	-	0.409	0.802	0.249	0.574	0.95	-0.493	-0.095	-0.561
		Expo	-1451.288	-0.409	-	0.393	-0.16	0.165	0.54	-0.902	-0.504	-0.97
		Expan	-1452.193	-0.802	-0.393	-	-0.554	-0.229	0.147	-1.295	-0.897	-1.363
	Lognormal	CS	-1450.918	-0.249	0.16	0.554	-	0.325	0.701	-0.741	-0.344	-0.809
		Expo	-1451.667	-0.574	-0.165	0.229	-0.325	-	0.376	-1.066	-0.669	-1.135
		Expan	-1452.532	-0.95	-0.54	-0.147	-0.701	-0.376	-	-1.442	-1.045	-1.51
	Expo	CS	-1449.212	0.493	0.902	1.295	0.741	1.066	1.442	-	0.397	-0.068
		Expo	-1450.127	0.095	0.504	0.897	0.344	0.669	1.045	-0.397	-	-0.466
		Expan	-1449.055	0.561	0.97	1.363	0.809	1.135	1.51	0.068	0.466	-
**A + B**	Strict	CS	-4259.814	-	0.166	-0.66	-20.18	-19.926	-20.5	-21.5	-22.09	-22.44
		Expo	-4260.197	-0.166	-	-0.826	-20.35	-20.093	-20.67	-21.67	-22.26	-22.6
		Expan	-4258.294	0.66	0.826	-	-19.52	-19.266	-19.84	-20.84	-21.43	-21.78
	Lognorma	CS	-4213.347	20.18	20.346	19.52	-	0.254	-0.321	-1.32	-1.91	-2.257
		Expo	-4213.932	19.926	20.093	19.266	-0.254	-	-0.575	-1.574	-2.164	-2.511
		Expan	-4212.609	20.501	20.667	19.841	0.321	0.575	-	-1	-1.589	-1.937
	Expo	CS	-4210.307	21.501	21.667	20.841	1.32	1.574	1	-	-0.59	-0.937
		Expo	-4208.95	22.09	22.256	21.43	1.91	2.164	1.589	0.59	-	-0.347
		Expan	-4208.15	22.438	22.604	21.778	2.257	2.511	1.937	0.937	0.347	-
