# Supplementary material for: Multiple introductions and recombination in Cryphonectria hypovirus 1: perspective for a sustainable biological control of chestnut blight
Source: Evol Appl. 2014 Apr 15;7(5):580–96. doi: 10.1111/eva.12157 (PMC4055179; doi:10.1111/eva.12157)
Supplement: Supplementary file 4 [file eva0007-0580-SD4.pdf]

Table S3: Estimation of substitution rates in *Cryphonectria hypovirus* 1 according to different population genetic models and on subsets of ORF A and ORF B alignments (ORF Ash and ORF Bsh).

**ORF-A$_{sh}$**	Population genetics model		
Clock model	Constant size (lower 95% HDP)	Exponential (lower 95% HDP)	Expansion (lower 95% HDP)
Strict	1.858E-04 (4.947E-05)	6.263E-04 (1,6401E-05)	1.859E-04 (7.154E-07)
Relaxed (Lognormal)	1.873E-04 (3.815E-05)	2.209E-04 (7,070E-05)	2.085E-04 (6.250E-07)
Relaxed (Exponential)	2.483E-04 (2.890E-05)	2.961E-04 (7.403E-05)	2.803E-04 (6.387E-07)

**ORF-B$_{sh}$**	Population genetics model		
Clock model	Constant size (lower 95% HDP)	Exponential (lower 95% HDP)	Expansion (lower 95% HDP)
Strict	1.283E-04 (3.536E-05)	1.434E-04 (5.178E-05)	7.318E-05 (1.167E-06)
Relaxed (Lognormal)	1.307E-04 (3.737E-05)	1.481E-04 (5.330E-05)	7.797E-05 (1.084E-06)
Relaxed (Exponential)	1.716E-04 (3.032E-05)	2.199E-04 (6.066E-05)	1.098E-04 (1.377E-06)

**ORF-A + ORF-B**	Population genetics model		
Clock model	Constant size (lower 95% HDP)	Exponential (lower 95% HDP)	Expansion (lower 95% HDP)
Strict	9.802E-05 (2.183E-05)	1.058E-04 (3.7548E-05)	3.019E-05 (1.652E-07)
Relaxed (Lognormal)	1.383E-04 (2.049E-05)	1.803E-04 (4.892E-05)	8.087E-05 (2.960E-07)
Relaxed (Exponential)	1.510E-04 (3.494E-05)	1.880E-04 (6.283E-05)	1.127E-04 (3.922E-07)
