# Supplementary material for: Multiple introductions and recombination in Cryphonectria hypovirus 1: perspective for a sustainable biological control of chestnut blight
Source: Evol Appl. 2014 Apr 15;7(5):580–96. doi: 10.1111/eva.12157 (PMC4055179; doi:10.1111/eva.12157)
Supplement: Supplementary file 5 [file eva0007-0580-SD5.docx]

**Data S1: Selection of the best –fitting model**

To select the best-fitting model, were calculated from the marginal likelihoods of competing models using Tracer v1.4 available from http://beast.bio.ed.ac.uk/Tracer. A significance threshold of 1.3 was set for log_10_(BF), which means that one model is about 20 times more likely to occur than the other (http://beast.bio.ed.ac.uk/Model_comparison). Each analysis was therefore repeated using constant size, exponential, logistic growth and bayesian skyline models in order to investigate the degree to which dating estimates are affected by the demographic model chosen. MCMC analysis was run for 25 million steps and sampled every 5,000 states. Length and number of MCMC chains were then adjusted so that the effective sample size for the root height parameter and other parameters was 200 (i.e. 25 to 50 million chains), indicating that the parameter space was sufficiently explored as suggested in Fargette et al. (2008). The convergence of the parameters to a stationary distribution was assessed with Tracer v1.4 and the statistical uncertainties were summarized in the 95% highest posterior density (HDP) intervals. The posterior distribution of evolutionary rate obtained from the heterochronous reference data set was subsequently incorporated as a prior distribution for the evolutionary rate in the ORF A_sh_ and ORF B_sh_ analyses, thereby placing a timescale on the histories of these strains and enabling an estimation of the time to the most recent common ancestor (tMRCA) for the strains (Drummond and Rambaut 2007).

**Literature cited:**

Drummond, A. J., and A. Rambaut. 2007. BEAST: Bayesian evolutionary analysis by sampling trees. *BMC Evolutionary Biology* 7:214.

Fargette, D., A. Pinel-Galzi, D. Sereme, S. Lacombe, E. Hebrard, O. Traore, and G. Konate. 2008. Diversification of Rice yellow mottle virus and related viruses spans the history of agriculture from the Neolithic to the present. *Plos Pathogens* 4(8): e1000125.

**Data S2: Positive selection analyses with CODEML**

CODEML’s models of codon substitution that assume positive selection (M2a and M8) were compared to null models (homogenous rate among sites, models M1a and M7). The model M1a assumes two site classes in proportions p_0_ and p_1_ (p_1_= 1-p_0_) and with rates ω_0_ and ω_1_ (with 0 < ω_0_ < 1 corresponding to purifying selection, and ω_1_ = 1, no selection). M2a adds an additional class of sites with ω_2_ as a free parameter with ω_2_ > 1 (positive selection) and in proportion p_2_. The neutral model M7 uses a beta distribution of sites within the interval 0 < ω < 1. The alternative model M8 adds an extra class of sites to the M7 model, allowing for positively selected sites with ω > 1. Model fittings were implemented with at least three different starting ω values (0.2, 1 and 2) as described in Joly et al. (2010). LRT were performed to compare 2ΔL, which is twice the difference in log likelihood ratio between the tested models, to a χ2 distribution. When the LRT is significant, this suggests that the alternative model fits the data better than the neutral model. Codon sites under positive selection were then identified using Bayes’ theorem to calculate the posterior probability that a particular amino acid belongs to a given selection class (neutral, deleterious or advantageous) under selected models (Yang 1997).

**Literature cited:**

Joly, D. L., N. Feau, P. Tanguay, and R. C. Hamelin. 2010. Comparative analysis of secreted protein evolution using expressed sequence tags from four poplar leaf rusts (*Melampsora* spp.). BMC Genomics 11:422.

Yang, Z. H. 1997. PAML: a program package for phylogenetic analysis by maximum likelihood. *Computer Applications in the Biosciences* 13 (5):555-556.

**Figure S1.** Nucleotide frequency and phylogenetic signal saturation analyses for the ORF-A and ORF-B genes of CHV1. Base frequencies across taxa (top) were calculated within 1st, 2nd, 3rd and all codon positions of each ORF. Phylogenetic signal saturation (bottom) is represented for each ORF by a plot of uncorrected distances (p-distances) and corrected distances for multiple substitutions (HKY85) between each pair of sequences.

**Figure S2.** Strict consensus from trees resulting from the maximum parsimony analyses (MP) of *Cryphonectria hypovirus* 1 ORF A and ORF B sequences. Tree statistics are presented in light blue boxes. Bootstrap support-values (of 1000 pseudoreplicates and ≥75%) are presented below branches. Diagrams depict the distribution of values found in MP homoplasy matrices.

**Figure S3.** Lineage divergence dates inferred from concatenated *Cryphonectria hypovirus* 1 sequences. The time-scale of evolutionary changes represented in the tree is indicated by the scale bar below it. Divergence dates with 95% CI are indicated above nodes; Bayesian posterior probabilities up to 0.5 are indicated below nodes.

**Figure S4.** Maximum clade credibility trees constructed from the ORF A_sh_ and ORF B_sh_ *Cryphonectria hypovirus* 1 datasets. The time-scale of evolutionary changes represented in the tree is indicated by the scale bar below it. Bayesian posterior probabilities are the same as in Fig. 4. Strain IDs are colored based on the region they were sampled. For each node ≥ 0.5 bayesian posterior probability, the upper graph represents the posterior location probabilities inferred from ORF A_sh_ or ORF B_sh_ datasets and the lower graph shows the posterior location probabilities obtained after 25 resamplings of ORF A_sh_ or ORF B_sh_ datasets (see Methods). Letters above columns indicate significant differences between posterior location probabilities; averages as determined by a Tukey’s HSD test after significant one-way ANOVA.
